# Curcumin and multiple health outcomes: critical umbrella review of intervention meta-analyses

**DOI:** 10.3389/fphar.2025.1601204

**Published:** 2025-06-05

**Authors:** Qin Xu, Hua Lian, Rui Zhou, Zhenzhen Gu, Jiao Wu, Yu Wu, Zhongyu Li

**Affiliations:** ^1^ Department of Oncology, Xiyuan Hospital, China Academy of Chinese Medical Sciences, Beijing, China; ^2^ Department of Oncology, The First Affiliated Hospital of Guizhou University of Traditional Chinese Medicine, Guiyang, Guizhou, China; ^3^ Department of Traditional Chinese Medicine Internal Medicine, Eye Hospital, China Academy of Chinese Medical Sciences, Beijing, China

**Keywords:** curcumin, metabolic indicators, health outcomes, evidence, meta-analysis, umbrella review

## Abstract

**Objective:**

This review aimed to determine the therapeutic effects and safety of oral curcumin compared with other comparators for human health and wellbeing outcomes.

**Methods:**

PubMed, Embase, and Cochrane Library were searched from their inception to 18 June 2024. The Assessment of Multiple Systematic Reviews-2 checklist, and Grading of Recommendations, Assessment, Development and Evaluation system were used to assess the methodological and evidence quality for each meta-analysis, respectively. The results are presented in a narrative review.

**Results:**

We included 25 studies. The overall methodological quality was relatively poor, and there is considerable room for improvement. The findings suggest that curcumin has potentially positive effects on lipid profiles, blood pressure, inflammatory markers and oxidative stress, musculoskeletal diseases, emotional and cognitive function, ulcerative colitis, liver and kidney function, primary dysmenorrhea or premenstrual syndrome, rheumatoid arthritis, COVID-19, painful statues, and HR-QOL. However, for many diseases, the conclusions remain uncertain.

**Conclusion:**

The available evidence suggests that curcumin is a safe medicinal agent that improves multiple clinical outcomes; however, the scientific quality of published studies needs to be improved.

## 1 Introduction

Curcumin, a natural compound derived from the rhizome of the turmeric plant (*Curcuma Longa*), has garnered significant attention for its health-promoting properties over the years (Ayub et al., 2024). It is particularly popular among residents of India and Southeast Asian countries (Kasprzak-Drozd et al., 2024). The present studies have found that curcumin has various pharmacological effects, including anti-inflammatory (Zhang et al., 2025), antioxidant (Nirgude et al., 2025), and immune response modulation (Gouda et al., 2024). These properties have promoted the exploration of its use as a potential drug for treating various chronic diseases. For instance, curcumin has demonstrated significant beneficial effects in musculoskeletal diseases (Jamali et al., 2020), gastrointestinal diseases (Ben-Horin et al., 2024), and mood or anxiety disorders (Asadi et al., 2020). Additionally, curcumin targets multitude signaling pathways and exerts cellular-level effects, making it a versatile supplement for various health conditions.

Specifically, curcumin’s antioxidant properties are attributed to scavenging free radicals and enhancing endogenous antioxidant defenses (Wang et al., 2024a). The anti-inflammatory benefits of curcumin are associated with pain reduction (Zhang et al., 2024) and mucosal protective effects in ulcerative colitis (Sadeghi et al., 2020; Wang et al., 2024b). Curcumin has been proven to inhibit cholesterol production and adipogenesis, thereby regulating lipid profiles and aiding in weight management (Shao et al., 2012). Furthermore, curcumin compounds have been shown to have actions similar to antidiabetic agents, reducing insulin resistance (Li et al., 2020), and interacting with the gut microbiota (Gu et al., 2024). Moreover, clinical practice guidelines have acknowledged the therapeutic value of curcumin, particularly in managing osteoarticular pain, making it among the most prescribed supplements for this condition (Liu et al., 2018; Mobasheri et al., 2024).

In recent decades, the popularity of curcumin supplements has surged, driven by widespread promoting in folk media, and the growing use of turmeric dietary supplements. In fact, turmeric has become the best-selling botanical dietary supplement in the United States (Panknin et al., 2023). The medicinal therapeutic value, diverse functionality, and rapid development of curcumin have driven considerable growth in the number of clinical trials worldwide (Yeung et al., 2019; Zhang et al., 2022). However, despite the abundance of clinical studies and mechanistic research supporting the health benefits of curcumin, there is a lack of high-quality integrated studies to determine which health effects are most strongly supported by evidence.

In evidence-based healthcare settings, systematic reviews and meta-analyses are vital for developing clinical practice guidelines and guiding clinical decision-making (Weissgerber, 2021). In particular, umbrella review serves as an effective method to assess the scientific quality of published systematic reviews and to summarize clinical evidence reported in domain-specific meta-analyses (Aromataris et al., 2015). This approach has been successfully applied to other natural products and dietary agents, including berberine (Li et al., 2023), anthocyanins (Sandoval-Ramírez et al., 2022), and tea (Keller and Wallace, 2021). Given the extensive research on curcumin’s health benefits and the need for a comprehensive synthesis of the available evidence, our review aimed to systematically identify and evaluate the therapeutic efficacy and safety of oral curcumin than any comparator for several human health and wellbeing outcomes. We hypothesize that curcumin will demonstrate significant therapeutic benefits across multiple health domains, supported by high-quality evidence from well-conducted systematic reviews and meta-analyses.

## 2 Materials and methods

The umbrella review is a novel method for deliberately searching, integrating and appraising available evidence on specific exposures and health outcomes among systematic reviews and/or meta-analyses (Aromataris et al., 2015). To provide a comprehensive evaluation of the therapeutic effect of curcumin, therefore, we only included systematic reviews with meta-analyses.

### 2.1 Screening and search strategy

We searched PubMed, Embase and the Cochrane Library from their inception to 18 June 2024, using medical subject headings and keywords, including “curcumin,” “turmeric,” “curcuma,” “curcuminoids,” “systematic reviews,” and “meta-analysis.” We only included English-language articles when searching these databases. Furthermore, we manually searched the references of the eligible articles to identify additional studies that may meet the inclusion criteria. The specific search methods are described in Supplementary Table S1. Two independent reviewers (Q.X. and J.W.) removed duplicates and screened the records based on titles and abstracts. Then, the potentially eligible records were downloaded for further evaluation. Any disagreements during the screening period, were resolved through discussion with a third reviewer (Y.W.).

### 2.2 Inclusion and exclusion criteria

The selected articles were eligible if they were meta-analyses that conducted a systematic review approach. The details of the inclusion criteria for our review were as follows: (1) Participants: adults aged ≥18 years, including patients and healthy participants; (2) interventions: curcumin alone or as a supplement on health outcomes; (3) controls: placebo, routine care, and others; (4) outcomes: any reported health outcomes, for example, metabolic indicators, gastrointestinal disorders, and musculoskeletal diseases; (5) study design: systematic review and meta-analyses based on randomized controlled studies (RCTs). We excluded preclinical studies, primary studies, genetic research, and conference abstracts. We also excluded studies on intravenous and topical administration, because of the potential differences in the mechanisms of action. If multiple meta-analyses were performed on the same intervention and outcome, we preferred the most recent, largest, and updated meta-analysis (Khan et al., 2019).

### 2.3 Data extraction

Two reviewers (Q.X. and R.Z.) independently extracted and cross-checked the data, including the first author, publication year, country, sample size, number of primary RCTs for meta-analysis, interventions, treatment duration, dosage, registration number, and health-related outcomes. In case of disagreements, the third reviewer (Z.Y.L.) was consulted for judgment.

### 2.4 Quality assessment of included studies

We referred to the relevant literature and used the Assessment of Multiple Systematic Reviews (AMSTAR)-2 checklist, the revised version of which was officially published by the AMSTAR Working Group in 2017, for methodological quality assessment (Shea et al., 2017). AMSTAR-2 checklist includes 16 items covering selecting topics, design, registration, data extraction, statistical analysis, and discussion of meta-analyses. Specifically, ‘Yes’ (Y), ‘Partial Yes’ (PY), or ‘No’ (N) were used to answer item-related questions. The AMSTAR-2 checklist classified the methodological quality into four levels: high, moderate, low, and critically low levels.

We used the Grading of Recommendations, Assessment, Development and Evaluation (GRADE) framework to assess the quality of evidence for each outcome (Guyatt et al., 2011). The GRADE system provides explicit criteria for grading the quality of evidence, including the risk of bias (RoB), inconsistency, indirectness, imprecision, and publication bias. We created an evidence map exhibiting the plausible benefits and the certainty of evidence for each intervention. The certainty of the evidence was assessed using the GRADE methodology (GRADEpro GDT) (https://gdt.gradepro.org/app/) and categorized evidence as high, moderate, low or very low credibility. Finally, the two reviewers (J.W. and Z.Z.G.) cross-checked the quality assessment and reached a consensus. Any disagreements were resolved by a third reviewer (Y.W.).

### 2.5 Statistical analysis

We extracted the necessary data (for example, estimated effects and 95% confidence interval (CI) for meta-analyses, p-values) directly from meta-analyses for narrative review. I^2^ statistics were used to assess heterogeneity between studies. Funnel plots, Egger’s test, and Begg’s test were used to assess publication bias. Statistical significance was set at two-sided *p* < 0.05.

## 3 Results

### 3.1 Findings of study screening

**FIGURE 1:**
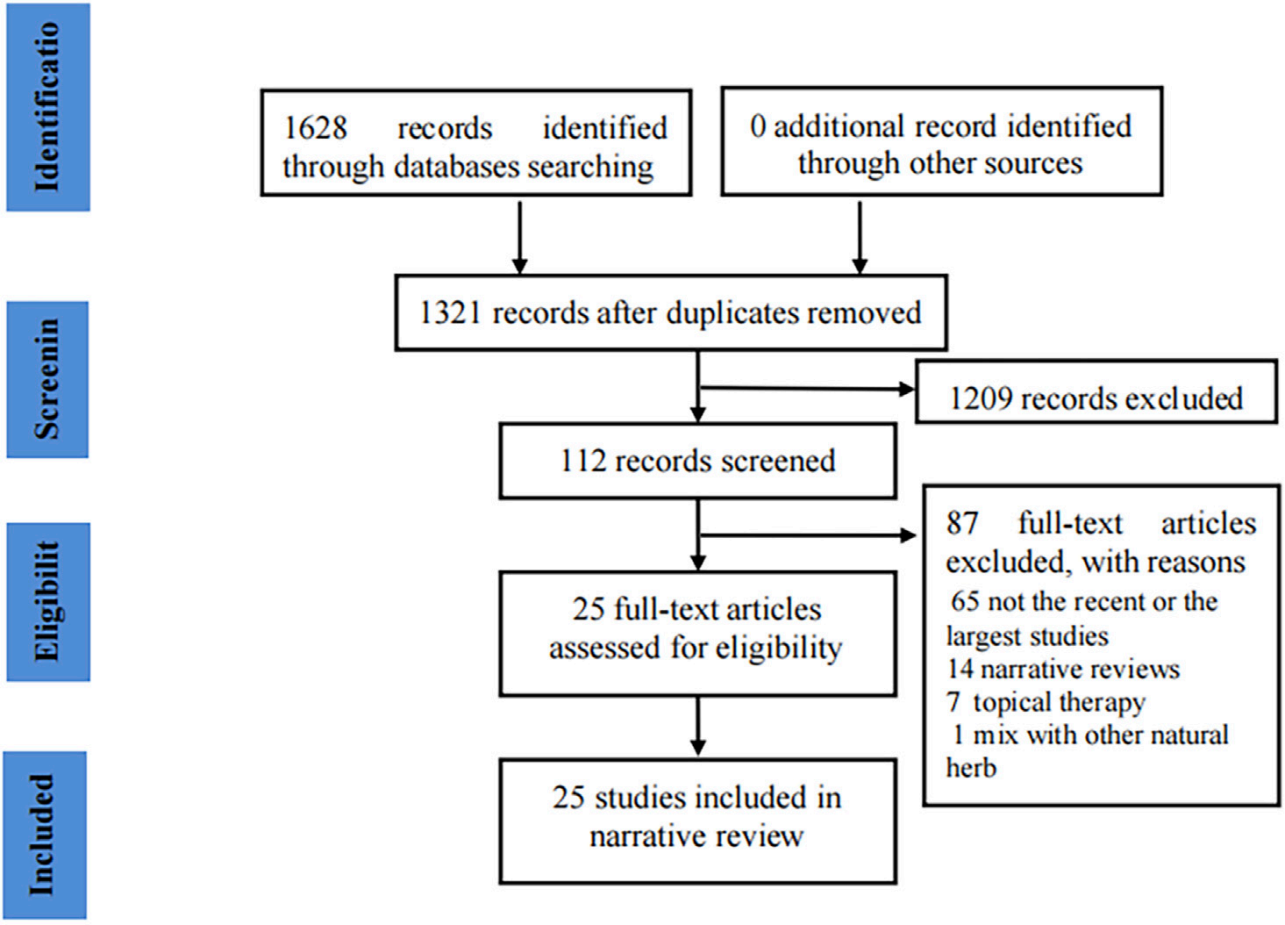
Flow chart for the included studies.

Initially, we obtained 1,628 records through a literature search. After removing 307 duplicates, 1,209 records were excluded by screening titles and abstracts. We screened the full-text of 112 meta-analyses and finally included 25 articles (Dehzad et al., 2023a; Dehzad et al., 2023d; Dehzad et al., 2023c; Dehzad et al., 2024; Ebrahimzadeh et al., 2024; Fathi et al., 2024; Sharifipour et al., 2024; Sahebkar and Henrotin, 2016; Ng et al., 2018; Sarraf et al., 2019; Zhu et al., 2019; Sadeghian et al., 2021; Wang et al., 2021a; Wang et al., 2021b; Zeng et al., 2021; Beba et al., 2022; Emami et al., 2022; Malekmakan et al., 2022; Shen et al., 2022; Tian et al., 2022; Yin et al., 2022; Dehzad et al., 2023b; Kou et al., 2023; Mirzaei Dahka et al., 2023; Shafiee et al., 2023) in our review (Figure 1 presents the flow chart of the study selection).

### 3.2 Research characteristics

Table 1 summarizes the characteristics of the included meta-analyses. The articles were published between 2016 and 2024. The meta-analyses were from six regions: 16 from Iran, six from China, and the remaining were from Australia, Singapore, and India. Only nine meta-analyses were registered on the PROSPERO platform and reported their registration number, but the remaining reviews failed to provide registration details. The number of primary clinical trials in the meta-analyses ranged from 3 to 66, and the number of participants ranged from 139 to 4,051. The Cochrane RoB assessment tool was the most commonly used to evaluate methodological quality, with one review each in the Jadad score and JBI Critical Appraisal Checklist, and two articles that did not report the tool used to assess methodological quality. Sixteen meta-analyses included only placebo-controlled trials, and the other meta-analyses included placebo, routine care, or medication as comparators. The curcumin dose varied considerably across the original studies, ranging from 50 to 6,000 mg. The treatment duration of curcumin interventions ranged from 1 day to 12 months in the meta-analyses.

**TABLE 1:** Characteristics of included studies.

Reference	Country	Health status	Number of primary studies	Sample size (I/C)	Interventions/comparations	Dose	Duration	Registration information	Bias of risk assessment	Health-related outcomes
Ebrahimzadeh et al. (2024)	Iran	NAFLD	21	1,191 (600/591)	Curcumin/placebo	50–3,000 mg/day	8–12 weeks	PROSPERO (CRD42023457942)	JBI Critical Appraisal Checklist	FBG, HbA1C, insulin levels, HOMA-IR, QUICKI, SBP, DBP, IL-6, CRP, TNF-α, Weight, WC, BMI
Dehzad et al. (2024)	Iran	Adults	35	2,250(NA)	Curcumin or turmeric supplementation/placebo	50–3,000 mg/day	4–24 weeks	PROSPERO (CRD42022353940)	Cochrane	SBP, DBP, VCAM-1, ICAM-1, FMD, and PWV
Fathi et al. (2024)	Iran	Adults with anxiety or have anxiety symptoms	8	567(NA)	Curcumin/placebo	80–1,000 mg/day	4–12 weeks	NA	Cochrane	Anxiety symptoms
Sharifipour et al. (2024)	Iran	Women with primary dysmenorrhea or PMS	5	379 (188/191)	Curcumin/placebo	200–1,500 mg/day	NA	NA	Cochrane	The severity of dysmenorrhea, PMS severity, behavioral symptoms of PMS, mood symptoms of PMS, and physical symptoms of PMS
Dehzad et al. (2023a)	Iran	Adults	64	4,051(NA)	Curcumin or turmeric supplementation/placebo	80–4,000 mg/day	4–24 weeks	PROSPERO (CRD42022353931)	Cochrane	TC, TG, LDL-C, HDL-C, Apo-A, and Apo-B
Dehzad et al. (2023d)	Iran	Adults	60	3,691(NA)	curcumin/turmeric supplementation/placebo	50–3,000 mg/day	4–36 weeks	PROSPERO (CRD42022350946)	Cochrane	BW, BMI, WC, BFP, leptin, and adiponectin
Dehzad et al. (2023c)	Iran	Adults	66	3,953(NA)	Curcumin or turmeric/placebo	80–3,000 mg/day	4–24 weeks	PROSPERO(CRD42022353946)	Cochrane	CRP, TNF-α, IL-6, IL-1β, TAC, MDA, and SOD
Kou et al. (2023)	India	RA	10	539(NA)	Curcumin/placebo or standard treatment	120–1,000 mg/day	3 weeks–3 months	PROSPERO (CRD42022361992)	Cochrane	ESR, CRP, DAS, RA, VAS, TJC, SJC
Dehzad et al. (2023b)	Iran	Adults	31	1948(NA)	Curcumin/placebo	80–3,000 mg/day	4–24 weeks	PROSPERO (CRD42022374871)	Cochrane	ALT, AST, GGT
Shafiee et al. (2023)	Iran	COVID-19	13	991(NA)	Curcumin/placebo or standard of care	40–525 mg/day	7–21 days	PROSPERO (CRD42022346913)	Cochrane	All-cause mortality, incidence of mechanical ventilation, incidence of hospitalization, rate of positive COVID-19 RT-PCR test, and rate of patients with no recovery
Mirzaei Dahka et al. (2023)	Iran	Breast cancer	4	NA	Curcumin/placebo	1,500–6,000 mg/day	3–7 weeks	NA	NA	RDS score
Tian et al. (2022)	China	T2DM	9	604 (284/281)	Curcumin/placebo or medication	80–2,100 mg/day	4 weeks–3 months	NA	Cochrane	TG, TC, LDL-C, and HDL-C, FBG and HbA1c
Shen et al. (2022)	China	PCOS	7	447(NA)	Curcumin/placebo or medication	80–1,500 mg/day	6 weeks–6 months	PROSPERO (CRD42022332394)	Cochrane	FBG, fasting insulin, HOMA-IR, 2-h glucose, 2-h insulin, HgbA1C, QUICKI, BMI, WHR, weight, WC, LH, FSH, LH/FSH, testosterone, FAI, DHEAS, TC, TG, HDL-C, LDL-C, CRP
Beba et al. (2022)	Iran	Adults	10	316(NA)	Curcumin/placebo	150 to 5,000 mg/day	1–56 days	NA	Cochrane	CK, activity, VAS score, inflammation, MVC and ROM
Emami et al. (2022)	Iran	CKD	10	523(NA)	Curcumin/control	80–250 mg/day	8–12 weeks	NA	NA	IL-6, TNF-α, hs-CRP
Yin et al. (2022)	China	UC	6	385(NA)	Curcumin/placebo	140–3,000 mg/day	4 weeks–6 months	No	Cochrane	Clinical remission, endoscopic remission, clinical improvement and endoscopic improvement
Malekmakan et al. (2022)	Iran	CKD	4	265 (138/127)	Turmeric/Curcumin/placebo	60–1,500 mg/day	15 days–4 months	NA	Cochrane	Proteinuria level
Wang et al. (2021b)	China	Adults with depression or have depressive symptoms	10	594 (327/267)	Curcumin/placebo	80–3,000 mg/day	4–24 weeks	No	Cochrane	Depressive symptoms, response rates
Zeng et al. (2021)	China	Osteoarthritis	15	1,621(NA)	Curcuma longa extracts and curcumin/placebo or conventional therapy	197–1,500 mg/day	4–12 weeks	NA	Cochrane	VAS and WOMAC score-pain, WOMAC-function, WOMAC-stiffness, score of OA and biochemical indicators
Wang et al. (2021a)	Australia	Knee Osteoarthritis	16	1810(NA)	Turmeric extracts/placebo or active comparators	80–200 mg/day	4–16 weeks	NA	Cochrane	Knee Pain, physical function, inflammatory biomarkers
Sadeghian et al. (2021)	Iran	NR	10	730 (355/375)	Curcumin/placebo	80–3,000 mg/day	1–12 months	NA	Cochrane	HR-QOL
Sarraf et al. (2019)	Iran	Adults	4	139 (69/70)	Curcumin/placebo	200–1,820 mg/day	8–12 weeks	No	Cochrane	BDNP
Zhu et al. (2019)	China	Older adults or individuals with AD or schizophrenia	5	289 (145/144)	Curcumin/placebo	32–4,000 g/day	4 weeks–12 months	NA	Cochrane	Cognition function, depression
Ng et al. (2018)	Singapore	IBS	3	326(NA)	Curcumin/placebo	60–5,000 mg/day	4–18 weeks	NA	Cochrane	IBS symptoms
Sahebkar and Henrotin (2016)	Iran	Adults with any pain	8	606(NA)	Curcuminoids or curcuminoid containing extracts/placebo or routine care	400–6,000 mg/day	4 days–8 weeks	NA	Jadad scale	Pain severity

Abbreviations: NA: not available; I: intervention; C: comparator; T2DM, Type 2 diabetes mellitus; HbA1c, hemoglobin A1c; NAFLD, nonalcoholic fatty liver disease; FBG: fasting blood glucose; HOMA-IR: homeostatic model assessment for insulin resistance; QUICKI: quantitative insulin-sensitivity check index; PCOS: polycystic ovarian syndrome; INS: insulin; OGTT: oral glucose tolerance test; SBP: systolic blood pressure; DBP: diastolic blood pressure; VCAM-1: vascular cell adhesion molecule-1; FMD: flow-mediated dilation; PWV: pulse wave velocity; TC: total cholesterol; TG: triglyceride; LDL-C: low-density-lipoprotein cholesterol; HDL-C: high-density lipoprotein cholesterol; Apo-A: apolipoproteins A; Apo-B: apolipoproteins B; BMI: body mass index; CRP: C-reactive protein; TNF-α: tumor necrosis factor-alpha; IL-6: interleukin-6; IL-1β: interleukin 1beta; IL-8: interleukin-8; RA: rheumatoid arthritis; TAC: total antioxidant capacity; MDA: malondialdehyde; SOD: superoxide dismutase; IBS: irritable bowel syndrome; BDNF: brain-derived neurotrophic factor; WOMAC: Western Ontario and McMaster Universities Osteoarthritis Index; VAS: visual analogue scale; ALT: alanine aminotransferase; AST: aspartate aminotransferase; GGT: gamma-glutamyltransferase; DHEA: dehydroepiandrosterone-sulfate; LH: luteinizing hormone; FSH: follicle-stimulating hormone; FAI: free androgen index; PMS: premenstrual syndrome.

### 3.3 Methodological quality

Most meta-analyses (n = 19, 76%) were classified as very low quality, with the remaining as low quality (n = 3, 12%) and moderate quality (n = 3, 12%). No articles were rated as high-quality. Table 2 provides the assessment results of the included meta-analyses.

**TABLE 2:** Quality appraisal results of included systematic reviews using the AMSTAR-2 Tool.

Citation	Item 1	Item 2	Item 3	Item 4	Item 5	Item 6	Item 7	Item 8	Item 9	Item 10	Item 11	Item 12	Item 13	Item 14	Item 15	Item 16	Overall rating
Ebrahimzadeh et al. (2024)	Y	Y	Y	Y	Y	Y	N	Y	PY	N	Y	Y	Y	Y	N	Y	Very low
Dehzad et al. (2024)	Y	Y	Y	Y	Y	Y	Y	Y	Y	N	Y	Y	N	Y	Y	Y	Moderate
Fathi et al. (2024)	Y	N	N	Y	N	Y	N	Y	Y	N	Y	Y	Y	Y	N	Y	Very low
Sharifipour et al. (2024)	Y	N	N	PY	Y	Y	N	Y	Y	N	Y	Y	N	Y	N	Y	Very low
Dehzad et al. (2023a)	Y	Y	Y	PY	Y	Y	Y	Y	Y	N	Y	Y	Y	Y	Y	Y	Moderate
Dehzad et al. (2023d)	Y	Y	N	Y	Y	Y	Y	Y	Y	N	Y	Y	Y	Y	Y	Y	Moderate
Dehzad et al. (2023c)	Y	Y	Y	Y	Y	Y	Y	Y	Y	N	Y	Y	N	Y	Y	Y	Low
Kou et al. (2023)	Y	Y	Y	Y	Y	Y	N	N	PY	N	Y	Y	N	Y	Y	Y	Very low
Dehzad et al. (2023b)	Y	Y	Y	Y	Y	Y	Y	Y	Y	N	Y	Y	N	Y	Y	Y	Low
Shafiee et al. (2023)	Y	Y	Y	Y	Y	Y	N	Y	Y	N	Y	Y	N	Y	N	Y	Critically low
Mirzaei Dahka et al. (2023)	Y	N	N	PY	Y	Y	N	Y	N	N	Y	Y	Y	N	Y	Y	Critically low
Tian et al. (2022)	Y	N	Y	Y	Y	Y	N	Y	Y	N	Y	Y	Y	Y	Y	Y	Critically low
Shen et al. (2022)	Y	Y	Y	Y	Y	Y	N	Y	Y	N	Y	Y	Y	N	N	Y	Critically low
Beba et al. (2022)	Y	N	N	PY	Y	N	Y	Y	Y	N	Y	Y	N	Y	Y	Y	Critically low
Emami et al. (2022)	N	N	N	PY	Y	Y	Y	N	N	N	Y	N	N	N	Y	Y	Critically low
Yin et al. (2022)	Y	N	N	PY	Y	Y	N	Y	Y	N	Y	Y	Y	Y	N	Y	Critically low
Malekmakan et al. (2022)	N	N	Y	PY	Y	Y	N	PY	Y	N	Y	N	N	N	Y	Y	Critically low
Wang et al. (2021b)	Y	PY	Y	Y	Y	Y	N	Y	Y	N	Y	Y	Y	Y	Y	Y	Critically low
Zeng et al. (2021)	Y	N	Y	PY	N	Y	Y	Y	Y	N	Y	N	N	Y	Y	Y	Critically low
Wang et al. (2021a)	Y	Y	N	Y	Y	Y	N	Y	Y	Y	Y	Y	N	Y	Y	Y	Critically low
Sadeghian et al. (2021)	N	N	N	Y	Y	N	Y	Y	Y	N	Y	Y	Y	Y	Y	Y	Low
Sarraf et al. (2019)	Y	PY	Y	PY	Y	Y	N	Y	Y	N	Y	N	Y	Y	Y	Y	Critically low
Zhu et al. (2019)	N	N	Y	PY	N	Y	Y	Y	Y	N	Y	N	N	Y	N	Y	Critically low
Ng et al. (2018)	Y	N	N	Y	Y	N	Y	N	Y	N	Y	N	Y	N	N	Y	Critically low
Sahebkar and Henrotin (2016)	N	N	Y	PY	N	N	N	Y	PY	N	Y	Y	Y	Y	Y	N	Critically low
Y (%)	20 (80%)	10 (40%)	15 (60%)	14 (56%)	21 (84%)	21 (84%)	14 (56%)	21 (84%)	20 (80%)	1 (4%)	25 (100%)	19 (76%)	13 (52%)	20 (80%)	17 (68%)	24 (96%)	
PY (%)	0	2 (8%)	0	11 (44%)	0	0	0	1 (4%)	3 (12%)	0	0	0	0	0	0	0	
N (%)	5 (20%)	13 (52%)	10 (40%)	0	4 (16%)	4 (16%)	11 (44%)	3 (12%)	2 (8%)	24 (96%)	0	6 (24%)	12 (48%)	5 (20%)	8 (32%)	1 (4%)	

Note: AMSTAR-2: Assessment of Multiple Systematic Reviews (AMSTAR)-2; Y: yes; N: no; P: partial yes. AMSTAR-2 consists of 16 items, seven of which (items 2, 4, 7, 9, 11, 13, and 15) are critical domains. Based on the critical and non-critical items, the overall methodological quality of each systematic review can be rated as high (no or one non-critical weakness), moderate (more than one non-critical flaw), low (one critical weakness or without a non-critical weakness), or critically low (more than one critical flaw or no non-critical weakness).

According to the GRADE, 82.79% (n = 101) of outcomes were rated as very low to low certainty, indicating limited confidence in the real word. The remaining 13.93% (n = 17) and 3.28% (n = 4) of the outcomes had moderate or high levels of confidence levels, respectively (Supplementary Table S2). The GRADE was impacted by the risk of bias, inconsistency (high heterogeneity), and imprecision (small sample size). Additionally, the limited number of available studies precluded a comprehensive assessment of publication bias, which could have exerted an indeterminate influence on the conclusions.

### 3.4 Therapeutic efficacy and safety of curcumin

**FIGURE 2:**
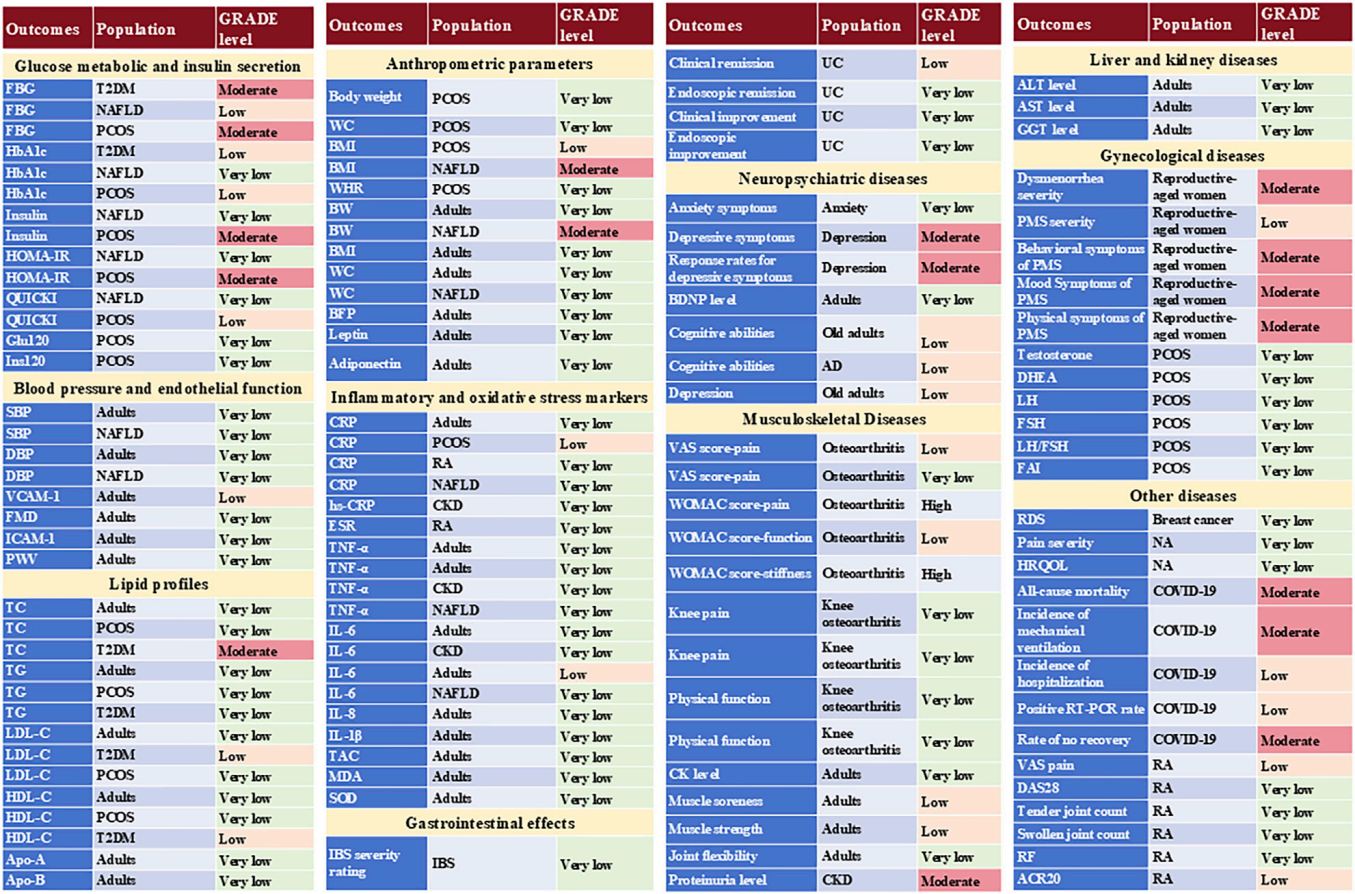
Summary of the evidence for the therapeutic effects of curcumin.

Supplementary Table S2 lists meta-analyses evaluating the effect of curcumin on each human outcome, as reported in the included systematic reviews. These results are more simply presented in Figure 2.

#### 3.4.1 Metabolic indicators

##### 3.4.1.1 Glucose metabolic and insulin secretion

Three meta-analyses have evaluated the effects of curcumin on glucose metabolism and insulin levels (Shen et al., 2022; Tian et al., 2022; Ebrahimzadeh et al., 2024). In patients with type 2 diabetes mellitus (T2DM), curcumin significantly reduced blood glucose levels (WMD: 8.85 mg/dL; 95%CI: 14.4, −3.29 mg/dL; I^2^ = 41.2%) and hemoglobin A1c (HbA1c) (WMD: 0.54%; 95%CI: 0.81, −0.27; I^2^ = 65.2%) when compared with control treatment (Tian et al., 2022). In patients with nonalcoholic fatty liver disease (NAFLD), the meta-analysis indicated that curcumin significantly reduced fasting blood glucose (FBG) (WMD: 2.83; 95%CI: 4.61, −1.06; I^2^ = 51.3%), homeostatic model assessment for insulin resistance (HOMA-IR) (WMD: 0.52; 95%CI: 0.84, −0.20; I^2^ = 82.8%), but not including HbA1c (WMD: 0.17; 95%CI: 0.44, 0.11, I^2^ = 92.4%), insulin levels (WMD: 0.14; 95%CI: 1.03, 0.76; I^2^ = 83.0%) and quantitative insulin-sensitivity check index (QUICKI) (WMD: 0.01; 95%CI: 0.00, 0.01; I^2^ = 96.2%) compared with placebo (Ebrahimzadeh et al., 2024). In patients with polycystic ovarian syndrome (PCOS), curcumin reduced FBG (WMD: 3.618; 95%CI: 5.165, −2.071; I^2^ = 20.4%), and insulin levels (WMD: 1.834; 95%CI: 2.701, −0.968; I^2^ = 8.4%) (Shen et al., 2022). Moreover, it observed improvement on QUICKI (WMD: 0.011, 95%CI: 0.005, 0.017; I^2^ = 39.6%) and HOMA-IR (WMD: 0.565; 95%CI: 0.779, −0.351; I^2^ = 0.0%), but not including blood glucose at 2 h after oral glucose tolerance test (OGTT) (WMD: 0.063, 95%CI: 2.307, 2.181; I^2^ = 87.4%), insulin at 2 h after OGTT (WMD: 12.445; 95%CI: 44.384, 19.494; I^2^ = 0.0%) and HbA1c (WMD = −0.042, 95%CI: 0.471, 0.387, I^2^ = 56.8%) (Shen et al., 2022).

##### 3.4.1.2 Blood pressure and endothelial function

Two meta-analyses evaluated the effects of curcumin on blood pressure and endothelial function (Dehzad et al., 2024; Ebrahimzadeh et al., 2024). For the effect on blood pressure, one meta-analysis demonstrated that curcumin/turmeric supplementation has a beneficial effect on regulating systolic blood pressure (SBP) (WMD: −2.02 mmHg; 95% CI: −2.85, −1.18; I^2^ = 96.7%), diastolic blood pressure (DBP) (WMD: −0.82 mmHg; 95% CI: −1.46, −0.18; I^2^ = 93.2%) in adult population when compared to placebo (Dehzad et al., 2024). However, another meta-analysis indicated that curcumin failed to reduce SBP (WMD: −0.93; 95% CI: −2.36, 0.50; I^2^ = 83.4%) and DBP (WMD: −1.37; 95% CI: −3.09, 0.35; I^2^ = 90.5%) in patients with NAFLD compared to placebo (Ebrahimzadeh et al., 2024). Regarding endothelial function, curcumin/turmeric supplementation reduced the levels of vascular cell adhesion molecule-1 (WMD: −39.19 ng/mL; 95% CI: −66.15, −12.23; I^2^ = 73%), and flow-mediated dilation (WMD: 2.00%; 95% CI: 1.07, 2.94; I^2^ = 79.5%), but did not significantly change ICAM-1 (WMD: 17.05 ng/mL; 95%CI: 80.79, 46.70; I^2^ = 94.1%), or pulse wave velocity (WMD: −79.53 cm/s; 95%CI: −210.38, 51.33; I^2^ = 99.7%) in the adult population compared to placebo (Dehzad et al., 2024).

##### 3.4.1.3 Lipid profiles

Three meta-analyses evaluated the effects of curcumin on lipid profiles (Shen et al., 2022; Tian et al., 2022; Dehzad et al., 2023a). Compared to placebo, one meta-analysis found that curcumin/turmeric supplementation improved lipid indices, including total cholesterol (TC) (WMD: 3.99 mg/dL; 95% CI: 5.33, −2.65; I^2^ = 97.0%), triglyceride (TG) (WMD: 6.69 mg/dL; 95% CI: 7.93, −5.45; I^2^ = 95.7%), low-density-lipoprotein cholesterol (LDL-C) (WMD: 4.89 mg/dL; 95% CI: 5.92, −3.87; I^2^ = 95.6%), and high-density lipoprotein cholesterol (HDL-C) (WMD:1.80 mg/dL; 95%CI:1.43, 2.17; I^2^ = 95.0%) in adults. However, it has non-significant effects on apolipoproteins-A (WMD:1.58 mg/dL; 95% CI: 3.49, 6.56, I^2^ = 64.4%) or apolipoproteins B (WMD:1.35 mg/dL; 95% CI: 9.74, 12.44; I^2^ = 83.4%) (Dehzad et al., 2023a). For patients with T2DM, compared to controls, curcumin significantly reduced TG (WMD: 18.97 mg/dL; 95%CI: 36.47, −1.47; I^2^ = 80.5%), TC (WMD: 8.91 mg/dL; 95% CI: 14.18, −3.63; I^2^ = 28.9%), but not including LDL-C (WMD: 4.01 mg/dL; 95% CI: 10.96, 2.95; I^2^ = 49.7%) and HDL-C (WMD: 0.32 mg/dL; 95%CI: 0.74, 1.37; I^2^ = 19.1%) (Tian et al., 2022). For patients with PCOS, curcumin significantly reduced TC (WMD: 15.591; 95% CI: 27.908, −3.273; I^2^ = 68.9%), but were not identified TG (WMD: 8.889; 95% CI: 27.246, 9.468; I^2^ = 91.5%), LDL-C (WMD: 6.427; 95%CI: 17.343, 4.489; I^2^ = 78.8%) and HDL-C (WMD:3.713; 95% CI: 0.786, 8.211; I^2^ = 81.3%) compared to the control group (Shen et al., 2022).

#### 3.4.2 Anthropometric measurements

Three meta-analyses evaluated the effect of curcumin on anthropometric measurements among different settings (Shen et al., 2022; Dehzad et al., 2023d; Ebrahimzadeh et al., 2024). Compared to placebo, we found that supplementation with curcumin/turmeric significantly reduced body weight (WMD: 0.82 kg; 95% CI: 1.30, −0.35; I^2^ = 78.7%), body mass index (BMI) (WMD: 0.30 kg/m^2^; 95% CI: 0.53, −0.06; I^2^ = 94.7%), waist circumference (WMD: 1.31 cm; 95%CI: 1.94, −0.69; I^2^ = 78.5%), body fat percentage (WMD: 0.88%; 95% CI: 1.51, −0.25; I^2^ = 86.2%), leptin (WMD: 4.46 ng/mL; 95%CI: 6.70, −2.21; I^2^ = 96.1%), and increased adiponectin (WMD:2.48 μg/mL; 95% CI: 1.34, 3.62; I^2^ = 96.3%) in adults (Dehzad et al., 2023d). In patients with NAFLD, curcumin supplementation also significantly reduced body weight (WMD: 0.81; 95% CI: 1.28, −0.35; I^2^ = 0.0%) and BMI (WMD: 0.35; 95% CI: 0.57, −0.13; I^2^ = 0.0%), but had no significant effect on waist circumference (WMD: 01.80; 95% CI: 3.61, 0.02; I^2^ = 87.2%) when compared to placebo (Ebrahimzadeh et al., 2024). Regarding PCOS patients, curcumin reduced BMI (WMD: 0.267; 95% CI: 0.450, −0.084; I^2^ = 0.0%) compared to controls. However, there were non-significant effects on body weight (WMD: 0.924; 95% CI: 2.009, 0.162; I^2^ = 45.2%), waist circumference (WMD: 1.475, 95%CI: 4.519, 1.570; I^2^ = 81.6%), and waist-hip ratio (WMD: 0.024; 95% CI: 0.048, 0.000; I^2^ = 0.0%) (Shen et al., 2022).

#### 3.4.3 Inflammatory and oxidative stress markers

Six meta-analyses evaluated the effect of curcumin on inflammatory and oxidative stress markers (Beba et al., 2022; Emami et al., 2022; Dehzad et al., 2023c; Kou et al., 2023; Shen et al., 2022; Ebrahimzadeh et al., 2024). Compared to placebo, curcumin/turmeric supplementation significantly reduced C-reactive protein (CRP) level (WMD: 0.58 mg/L; 95%CI: 0.74, −0.41; I^2^ = 98.9%), tumor necrosis factor-alpha (TNF-α) level (WMD: 3.48 pg/mL; 95%CI: 4.38, −2.58; I^2^ = 99.4%), interleukin-6(IL-6) level (WMD: 1.31 pg/mL; 95%CI: 1.58, −0.67; I^2^ = 88.2%) but failed to affect interleukin 1beta (IL-1β) level (WMD: 0.46 pg/mL; 95% CI: 1.18, 0.27; I^2^ = 75.8%) in adults (Dehzad et al., 2023c). In adult with delayed-onset muscle soreness, curcumin supplementation reduced TNF-α levels compared to placebo (WMD: 0.22 pg/mL; 95%CI: 0.33, −0.10; I^2^ = 93.2%), but did not affect IL-6 (WMD: 0.05 pg/mL; 95% CI: 0.14, 0.04; I^2^ = 46.9%) and interleukin-8 (IL-8) levels (WMD: 0.33 pg/mL; 95% CI: 1.39, 0.73; I^2^ = 85.6%) (Beba et al., 2022). For patients with PCOS, curcumin reduced CRP levels (WMD: 0.785; 95%CI: 1.553, −0.017; I^2^ = 23.9%) compared to placebo (Shen et al., 2022). In patients with rheumatoid arthritis (RA), curcumin significantly reduced the ESR (MD: 29.47; 95%CI: 54.05, −4.88; I^2^ = 99%) and CRP levels (MD: 0.93; 95%CI: 1.33, −0.53; I^2^ = 89%) as compared to the control group (Kou et al., 2023). In chronic kidney disease receiving hemodialysis, when compared with placebo, curcumin had a non-significant effect on IL-6 (SMD:0.24%; 95% CI: 0.14, 0.62; I^2^ = 97.1%), TNF-a (SMD:0.11; 95% CI: 0.19, 0.40; I^2^ = 95.9%) or hs-CRP (SMD: 0.17%; 95%CI: 0.36, 0.03; I^2^ = 79.6%) (Emami et al., 2022). However, compared with placebo, curcumin supplementation had a non-significant effect on IL-6 (WMD: 1.67; 95%CI: 3.80, 0.47; I^2^ = 81.3%), TNF-a (WMD: 2.58; 95%CI: 6.21, 1.06; I^2^ = 98.6%) and CRP levels (WMD: 2.59; 95% CI: 5.45, 0.26; I^2^ = 99.4%) in patients with NAFLD (Ebrahimzadeh et al., 2024).

Regaarding antioxidants, intake of curcumin/turmeric supplementation significantly increased total antioxidant capacity (WMD:0.21 mmol/L; 95% CI: 0.08, 0.33; I^2^ = 99.6%), and decreased malondialdehyde levels (WMD: 0.33 μmol/L; 95%CI: 0.53, −0.12; I^2^ = 99.6%) and superoxide dismutase activity (WMD:20.51 u/L; 95%CI: 7.35, 33.67; I^2^ = 95.4%) (Dehzad et al., 2023c).

#### 3.4.4 Gastrointestinal disorders

Two meta-analyses have explored the effects of curcumin on gastrointestinal disorders (Ng et al., 2018; Yin et al., 2022). For patients with irritable bowel syndrome (IBS), curcumin had a beneficial albeit not statistically significant effect on IBS severity ratings compared to placebo (SMD: 0.466; 95% CI: 1.113, 0.182; I^2^ = 85.22%) (Ng et al., 2018). Regarding patients with ulcerative colitis, adjuvant curcumin therapy was effective in inducing clinical remission (RR: 2.10; 95%CI: 1.13, 3.89; I^2^ = 80%), but not in inducing clinical improvement (RR: 1.62; 95% CI: 1.00, 2.61; I^2^ = 64%), endoscopic remission (RR: 4.17; 95% CI: 0.63, 27.71; I^2^ = 80%), and endoscopic improvement (RR: 4.13; 95% CI:0.20, 87.07; I^2^ = 79%) (Yin et al., 2022).

#### 3.4.5 Neuropsychiatric diseases

Four meta-analyses have explored the effects of curcumin on neuropsychiatric diseases (Sarraf et al., 2019; Zhu et al., 2019; Wang et al., 2021b; Fathi et al., 2024). The meta-analysis indicated that compared to placebo, curcumin might contribute to alleviating anxiety symptoms (SMD: 1.56; 95%CI: 2.48, −0.64; I^2^ = 95.6%) (Fathi et al., 2024), reduce depressive symptoms (SMD: 0.32; 95%CI: 0.50, −0.13; I^2^ = 15%), and improve clinical response rates (OR: 3.20; 95% CI: 1.28, 7.99; I^2^ = 35%) in patients with psychological disorders (Wang et al., 2021b). One study analyzed the effects of curcumin on neurotransmitters. The results suggested that curcumin significantly increased the serum brain-derived neurotrophic factor (BDNF) levels in adults (WMD:1789.38 pg/mL; 95%CI: 722.04, 2,856.71; I^2^ = 83.5%)compared to the placebo (Sarraf et al., 2019). In older adults who received curcumin, cognitive function (SMD: 0.33; 95% CI:0.05, 0.62; I^2^ = 0%)was significantly improved, but not for depression (SMD: 0.29; 95% CI: 0.64, 0.05; I^2^ = 0%) compared to placebo (Zhu et al., 2019). However, in patients with Alzheimer’s disease, there was a trend towards worse performance in cognitive status (SMD: 0.90; 95%CI: 1.48, −0.32; I^2^ = 0%) when treated with curcumin compared to placebo (Zhu et al., 2019).

#### 3.4.6 Musculoskeletal diseases

Three meta-analyses have evaluated the effects of curcumin on the musculoskeletal diseases (Wang et al., 2021a; Zeng et al., 2021; Beba et al., 2022). In patients with osteoarthritis, compared with placebo, *Curcuma longa* extract and curcumin reduced visual analog scale (VAS) (WMD: 11.55; 95%CI: 14.3, −9.06; I^2^ = 0%) and Western Ontario and McMaster Universities Osteoarthritis Index (WOMAC) scores-pain (SMD: 0.66, 95% CI: 0.88, −0.43; I^2^ = 34%), WOMAC scores-function (SMD: 0.79; 95% CI: 1.27, −0.31; I^2^ = 75%), and WOMAC scores-stiffness (SMD: 0.35; 95%CI: 0.57, −0.12; I^2^ = 26%) (Zeng et al., 2021). When compared to non-steroidal anti-inflammatory drugs (NSAIDs), *Curcuma longa* extract and curcumin had similar effects on joint pain (WMD: 0.34; 95%CI: 1.25, 0.57; I^2^ = 0%) (Zeng et al., 2021). In knee osteoarthritis, compared to placebo, turmeric extract also significantly reduced knee pain (SMD: 0.82; 95%CI: 1.17, −0.47; I^2^ = 86.23%) and improved physical function (SMD: 0.75; 95% CI: 1.18, −0.33; I^2^ = 90.05%), but had similar effects as NSAIDs (Wang et al., 2021a). Furthermore, one meta-analysis evaluated the effects on adults with delayed-onset muscle soreness, the results found that curcumin supplementation reduced creatine kinase activity level (WMD: 65.98IU/L; 95% CI: 99.53, −32.44; I^2^ = 86.8%), and muscle soreness (WMD: 0.56; 95%CI: 0.84, −0.27; I^2^ = 61.2%) compared to the placebo group (Beba et al., 2022). Moreover, curcumin supplementation also significantly improved muscle strength (WMD:3.10 nm; 95% CI:1.45, 4.75; I^2^ = 0.0%) and affected joint flexibility (WMD: 6.49°, 95% CI: 3.91, 9.07; I^2^ = 71.7%) (Beba et al., 2022).

#### 3.4.7 Liver and kidney diseases

Two studies evaluated the effects of curcumin on liver and kidney functions (Malekmakan et al., 2022; Dehzad et al., 2023b). One meta-analysis suggested that oral turmeric supplementation significantly reduced the proteinuria levels (SMD: 0.72; 95% CI: 1.10, −0.35; I^2^ = 46.2%) in patients with chronic kidney disease compared to placebo (Malekmakan et al., 2022). Regarding liver function, curcumin/turmeric supplementation reduced blood alanine aminotransferase level (WMD: 4.09 U/L, 95%CI: 6.49, −1.70; I^2^ = 95.8%) and aspartate aminotransferase level (WMD: 3.81 U/L; 95%CI: 5.71, −1.91; I^2^ = 96.3%) but not gamma-glutamyltransferase level (WMD: 12.78 U/L; 95%CI: 28.20, 2.64; I^2^ = 98.0%) in adults (Dehzad et al., 2023b).

#### 3.4.8 Gynecological disorders

Two meta-analyses evaluated the effects of curcumin on gynecological disorders (Shen et al., 2022; Sharifipour et al., 2024). Regarding patients with PCOS, curcumin had a non-significant effect on improving testosterone (T) level (WMD: 0.128; 95%CI: 0.383, 0.127; I^2^ = 98.6%), dehydroepiandrosterone-sulfate (WMD: 8.239; 95% CI: 30.260, 13.781; I^2^ = 62.3%), luteinizing hormone (LH) (WMD: 0.003; 95%CI: 0.007, 0.000; I^2^ = 0.0%) and follicle-stimulating hormone (FSH) (WMD: 0.002; 95%CI: 0.024, 0.029; I^2^ = 0.0%) compared to placebo (Shen et al., 2022). Compared to the control group, there failed to observed that curcumin improve LH/FSH (WMD: 0.114; 95%CI: 0.311, 0.084; I^2^ = 0.0%) and ameliorating free androgen index (WMD: 0.245; 95% CI: 1.138, 0.647; I^2^ = 30.0%) (Shen et al., 2022). As for reproductive-aged women with primary dysmenorrhea or premenstrual syndrome (PMS), curcumin intake significantly reduced the severity of dysmenorrhea (MD: 1.25; 95% CI: 1.52, −0.98; I^2^ = 31%) and the overall score of PMS (SMD: 1.41; 95% CI: 1.81, −1.02; I^2^ = 0%) than placebo (Sharifipour et al., 2024). Furthermore, curcumin also significantly reduced behavioral symptoms (MD: 12.90; 95% CI: 17.82, −7.99; I^2^ = 0%), mood symptoms (MD: 17.61; 95% CI: 22.75, −12.46; I^2^ = 0%) and physical disorders (MD: 19.65; 95% CI: 25.50, −13.80; I^2^ = 0%) of PMS patients (Sharifipour et al., 2024).

#### 3.4.9 Other diseases

In patients with COVID-19, curcumin reduced the risk of all-cause mortality (RR: 0.37; 95% CI: 0.21, 0.65; I^2^ = 0%), and patients with no recovery status (RR: 0.55; 95% CI: 0.43, 0.69; I^2^ = 0%) but did no effect on the incidence of mechanical ventilation (RR: 0.23; 95% CI: 0.05, 1.07; I^2^ = 0%), hospitalization (RR: 0.17; 95% CI: 0.02, 1.40; I^2^ = 0%), and the rate of a positive viral polymerase chain reaction test (RR: 0.55; 95% CI: 0.40, 0.77; I^2^ = 32%) when compared to the control group (Shafiee et al., 2023). For RA, curcumin was beneficial for DAS28 (MD: 1.20; 95% CI: 1.85, −0.55; I^2^ = 92%), rheumatoid factor (MD: 24.15; 95% CI: 36.47, −11.83; I^2^ = 97%), VAS pain (MD: 5.32; 95% CI: 9.42, −1.22; I^2^ = 19%), swollen joint count (MD: 5.33; 95% CI: 9.90, −0.76; I^2^ = 98%) and tender joint count (MD: 6.33; 95%CI: 10.86, −1.81; I^2^ = 98%) compared to control group, but not including ACR-20 (MD:0.96; 95% CI:0.39, 1.52; I^2^ = 0%) (Kou et al., 2023). Moreover, one meta-analysis assessed the therapeutic effect of curcumin on the severity of radiation dermatitis in patients with breast cancer, and the results indicated that curcumin supplementation significantly reduced the radiation dermatitis severity score compared to the placebo group (WMD: 0.50; 95% CI: 0.72, −0.27; I^2^ = 95.7%) (Mirzaei Dahka et al., 2023). Regarding analgesic effects, one meta-analysis suggested that curcumin significantly reduced the pain severity (SMD: 0.57; 95%CI: 1.1, −0.03; I^2^ = 86%) in patients with painful statues (Sahebkar and Henrotin, 2016). Furthermore, oral curcumin had a strong positive impact on HR-QOL (SMD: 2.46; 95% CI: 1.30, 3.63; I^2^ = 97.4%) compared with placebo (Sadeghian et al., 2021).

### 3.5 Safety

Eight studies reported adverse events (Zhu et al., 2019; Wang et al., 2021a; Wang et al., 2021b; Zeng et al., 2021; Malekmakan et al., 2022; Shen et al., 2022; Yin et al., 2022; Kou et al., 2023), including gastrointestinal symptoms, such as bloating, nausea, abdominal pain, diarrhea and constipation, and other symptoms including headache, dizziness, rash and hot flushes. However, there were no serious adverse events.

## 4 Discussion

To our knowledge, this is the first review to assess the methodological quality and evidence of available meta-analyses on curcumin. Our review included 25 studies that evaluated the therapeutic and preventive effects of curcumin on diverse diseases. The findings suggested that curcumin has potential effects on lipid profiles, blood pressure, inflammatory markers and oxidative stress, musculoskeletal diseases, emotional and cognitive function, ulcerative colitis, liver and kidney function, primary dysmenorrhea or PMS, RA, COVID-19, and painful statues as well as HR-QOL. However, for many of the diseases the conclusions are still uncertain.

The biological activity of curcumin has been well confirmed, and it is expected to increase the clinical applicability of curcumin by revealing its mechanism of action in different diseases. In clinical trials, curcumin as an effective antihyperglycaemic agent, has been found to improve insulin resistance and reduce insulin and blood glucose levels. Numerous studies have revealed that curcumin induces PPAR-γ activation to regulate glucose metabolism (Jiménez-Flores et al., 2014). Additionally, studies revealed that curcumin could prevent hyperglycemia by promoting insulin secretion, improving β-cell function, and inhibiting β-cell apoptosis (Gu et al., 2024). Curcumin/turmeric can reduce blood pressure by inducing eNOS protein expression, enhancing antioxidant capacity by restoring glutathione, and decreasing the overproduction of reactive oxygen species (Ramaswami et al., 2004; Nakmareong et al., 2011). Curcumin/turmeric can also improve endothelial vasorelaxation response to acetylcholine, increase NO bioavailability, and induce several antioxidant enzyme genes expressions through activation of the Nrf2-antioxidant response element signaling pathways (Aggarwal and Sung, 2009; Rungseesantivanon et al., 2010; Suphim et al., 2010). The exact mechanism by which curcumin/turmeric may affect body measurements has not been fully determined. However, curcumin’s effects on anthropometric aspects have been associated with downregulation of the Janus kinase enzyme and inhibition of adipocyte differentiation (Dehzad et al., 2023d). Curcumin is effectively used in obesity treatment because it is a lipophilic molecule that rapidly penetrates cell membranes and may be associated with lipid metabolism, gut microbiota and anti-inflammatory potential (Kasprzak-Drozd et al., 2022).

A recent study found that curcumin exerts beneficial effects on gastrointestinal disorders. This may be related to the regulation of the ‘brain-gut axis’ and restoration of the integrity of the intestinal mucosal barrier (Yu et al., 2015; Wang et al., 2017). Notably, curcumin has been found to modulate neurotransmitters in the brain, particularly serotonin, dopamine and norepinephrine, which may account for its antidepressant effects (Spanoudaki et al., 2024). Curcumin also improves cognitive function. The neuroprotective properties of curcumin act by inducing cAMP response element-binding protein and, subsequently, BDNF activation (Gomez-Pinilla and Nguyen, 2012). These interactions may contribute to mental and neurological health.

Curcumin has potential therapeutic effects on bone, joint and muscle disorders. This may help modulate inflammatory processes and metabolic pathways, thereby reducing symptoms and potentially slowing disease progression (Maouche et al., 2024). Curcumin, a promising antiviral drug for COVID-19, has been revealed to have high inhibitory activity against this virus (Al-Doori et al., 2021). Meanwhile, computer simulations and molecular docking indicated that the monomer has a good ability to bind to coronavirus and host targets, thus blocking the virus-host binding site (Jena et al., 2021). Therefore, the use of curcumin as an antiviral and anti-inflammatory substance may improve the containment of the damage caused by COVID-19 patients.

Curcumin has potential protective effects on the liver and kidney functions. The mechanism by which curcumin attenuates proteinuria can be explained by referring to recent investigations regarding its anti-inflammatory enhanced autophagy effects (Fan et al., 2020). Given that curcumin is a potent antioxidant, its protective effect on liver function may be related to its free radical scavenging properties. The therapeutic effects of curcumin in radiodermatitis have been associated with anti-inflammatory and antioxidant properties, as well as the ability to stimulate the regeneration of skin epithelial cells and promote wound healing (Kasprzak-Drozd et al., 2024). Curcumin is widely used in most countries globally. Given its multidirectional effects, it is used for health-promoting purposes. Moreover, further mechanistic studies are needed to explore the effects of curcumin on various signaling cascades in the body.

Although many experts believe that natural remedies may be safer than conventional medicine, patients are still susceptible to adverse reactions to other ingredients. There is a need for certainty when combined with other therapies. Moreover, suitable dosages and contraindications of curcumin need to be explored. There is also a need to study the mechanisms that have revealed the mechanism of action of curcumin on different diseases. There is also a need to explore the mechanism underlying the effects of curcumin on different diseases.

Since the methodological quality assessed using the AMSTAR-2 checklist and the certainty of the outcome effects assessed using the GRADE grading were mostly very low to low, there is a need to improve the quality of future studies. We recommend that researchers make their studies public in advance. This could encourage researchers to comply with the protocol and reduce various biases (Sideri et al., 2018). It can also avoid unnecessary duplication and optimize limited resources (Booth et al., 2011). The protocol could be registered and published on platforms such as PROSPERO (https://www.crd.york.ac.uk/prospero/), OSF REGISTRIES (https: //osf.io/registries? viewonly=). The included primary studies are an important guarantee for the evaluation of evidence, and comprehensive search strategies, as well as reasonable inclusion criteria, an important guarantee for reliability. To improve the accuracy of the meta-analysis results, the following suggestions are made: Choosing multiple search methods and databases, tracing back references, retrieving registry information, consulting with experts in the relevant fields, and searching grey literature to ensure that the relevant literature is not missed.

Furthermore, it is necessary to explain the rationale and reasons for the inclusion of studies, which can provide reviewers and readers with a clearer understanding of whether the processes involved were justified. It is also important to note that authors need to provide a list of excluded literature and reasons, thus avoiding bias in meta-analyses. Furthermore, commercially funded studies are more likely to reach conclusions in favor of the sponsor’s product than independently funded studies (DeAngelis and Fontanarosa, 2008). Therefore, authors must keep detailed records of the funding sources for each study facilitate the judgement of whether funding could lead to a conflict of interest. The investigation and discussion of potential publication bias for the meta-analysis included in this overview needs to be improved. The funnel plot, Egger’s test, Begg’s test, and Macaskill’s can all be used to detect publication bias (Hayashino et al., 2005). Consequently, it is important to strengthen the quality of the systematic reviews to make more confident recommendations. Hence, future reviews should be rigorously reported following the PRISMA guidelines (Page et al., 2021) and use the best practice methods. Finally, given the heterogeneities and the inconsistencies, we suggest that future studies focus on resolving the existing ambiguities concerning the impact of turmeric/curcumin on health outcomes and clinical biomarkers in the high-quality human trials.

### 4.1 Strengths and limitations

This umbrella review has some strengths and limitations. For instance, our review synthesizes evidence based on clinical practice, and the findings improve our knowledge of the validity of curcumin in clinical settings. It is worth highlighting that we used a rigorous study design, including use the latest versions of AMSTAR-2 and the GRADE system, to assess methodological quality and quality of evidence. As a result, we synthesized up-to-date comprehensive evidence, which will help guide the integration of adjunctive curcumin use into clinical practice to address general health and wellbeing, as well as therapeutic disease management. For example, healthcare experts may recommend that patients take curcumin as needed to prevent or treat diseases. However, there remains a gap betwwenn evidence and clinical practice, and future research should explore the reasons and mechanisms in different populations. Most of the studies failed to register their protocols, which could impair the transparency and credibility of the evidence. Second, we searched only English databases, which may have limited access to some available evidence. We obtained evidence synthesized by existing reviews, where details of the original trials may have been omitted, and studies included in different reviews may have overlapped. Third, there was significant heterogeneity regarding participants, interventions and assessment of outcomes, which may have affected the stability and accuracy of the findings. Finally, not all included studies were of high quality, which could introduce potential bias.

## 5 Conclusion

This umbrella review provides up-to-date evidence for the effect of curcumin on diverse clinical outcomes in humans. Oral curcumin has been found to be safe and therapeutic for human health and wellbeing, with potential benefits for osteoarthritis, blood sugar, lipids, and blood pressure. Curcumin has also been associated with improvements in dysmenorrhea and polycystic ovary syndrome; inflammatory status, including RA, COVID-19, and radiation dermatitis; liver and kidney function; and gastrointestinal and psychological disorders. In summary, while curcumin has demonstrated potential therapeutic benefits across various health domains, its clinical application is still fraught with challenges. In the future, more high-quality studies are needed to determine the effects of curcumin on different populations and to determine the availability of personalized, effective interventions to optimize curcumin use in clinical and healthcare settings.

## Data Availability

The raw data supporting the conclusions of this article will be made available by the authors, without undue reservation.

## References

[B1] Aggarwal B. B. Sung B. (2009). Pharmacological basis for the role of curcumin in chronic diseases: an age-old spice with modern targets. Trends Pharmacol. Sci. 30 (2), 85–94. 10.1016/j.tips.2008.11.002 19110321

[B2] Al-Doori A. Ahmed D. Kadhom M. Yousif E. J. (2021). Herbal medicine as an alternative method to treat and prevent COVID-19. Baghdad J. Biochem. Appl. Biol. Sci. 2 (01), 1–20. 10.47419/bjbabs.v2i01.25

[B3] Aromataris E. Fernandez R. Godfrey C. M. Holly C. Khalil H. Tungpunkom P. (2015). Summarizing systematic reviews: methodological development, conduct and reporting of an umbrella review approach. Int. J. Evid. Based Healthc. 13 (3), 132–140. 10.1097/XEB.0000000000000055 26360830

[B4] Asadi S. Gholami M. S. Siassi F. Qorbani M. Sotoudeh G. (2020). Beneficial effects of nano-curcumin supplement on depression and anxiety in diabetic patients with peripheral neuropathy: a randomized, double-blind, placebo-controlled clinical trial. Phytother. Res. 34 (4), 896–903. 10.1002/ptr.6571 31788880

[B5] Ayub H. Islam M. Saeed M. Ahmad H. Al-Asmari F. Ramadan M. F. (2024). On the health effects of curcumin and its derivatives. Food Sci. Nutr. 12 (11), 8623–8650. 10.1002/fsn3.4469 39620006 PMC11606848

[B6] Beba M. Mohammadi H. Clark C. C. T. Djafarian K. (2022). The effect of curcumin supplementation on delayed-onset muscle soreness, inflammation, muscle strength, and joint flexibility: a systematic review and dose-response meta-analysis of randomized controlled trials. Phytother. Res. 36 (7), 2767–2778. 10.1002/ptr.7477 35574627

[B7] Ben-Horin S. Salomon N. Karampekos G. Viazis N. Lahat A. Ungar B. (2024). Curcumin-QingDai combination for patients with active ulcerative colitis: a randomized, double-blinded, placebo-controlled trial. Clin. Gastroenterol. Hepatol. 22 (2), 347–356.e6. 10.1016/j.cgh.2023.05.023 37302449

[B8] Booth A. Clarke M. Ghersi D. Moher D. Petticrew M. Stewart L. (2011). An international registry of systematic-review protocols. Lancet 377 (9760), 108–109. 10.1016/S0140-6736(10)60903-8 20630580

[B9] DeAngelis C. D. Fontanarosa P. B. (2008). Impugning the integrity of medical science: the adverse effects of industry influence. JAMA 299 (15), 1833–1835. 10.1001/jama.299.15.1833 18413880

[B10] Dehzad M. J. Ghalandari H. Amini M. R. Askarpour M. (2023a). Effects of curcumin/turmeric supplementation on lipid profile: a GRADE-assessed systematic review and dose-response meta-analysis of randomized controlled trials. Complement. Ther. Med. 75, 102955. 10.1016/j.ctim.2023.102955 37230418

[B11] Dehzad M. J. Ghalandari H. Amini M. R. Askarpour M. (2023b). Effects of curcumin/turmeric supplementation on liver function in adults: a GRADE-assessed systematic review and dose-response meta-analysis of randomized controlled trials. Complement. Ther. Med. 74, 102952. 10.1016/j.ctim.2023.102952 37178581

[B12] Dehzad M. J. Ghalandari H. Askarpour M. (2024). Curcumin/turmeric supplementation could improve blood pressure and endothelial function: a grade-assessed systematic review and dose-response meta-analysis of randomized controlled trials. Clin. Nutr. ESPEN 59, 194–207. 10.1016/j.clnesp.2023.12.009 38220376

[B13] Dehzad M. J. Ghalandari H. Nouri M. Askarpour M. (2023c). Antioxidant and anti-inflammatory effects of curcumin/turmeric supplementation in adults: a GRADE-assessed systematic review and dose-response meta-analysis of randomized controlled trials. Cytokine 164, 156144. 10.1016/j.cyto.2023.156144 36804260

[B14] Dehzad M. J. Ghalandari H. Nouri M. Askarpour M. (2023d). Effects of curcumin/turmeric supplementation on obesity indices and adipokines in adults: a grade-assessed systematic review and dose-response meta-analysis of randomized controlled trials. Phytother. Res. 37 (4), 1703–1728. 10.1002/ptr.7800 36882287

[B15] Ebrahimzadeh A. Mohseni S. Safargar M. Mohtashamian A. Niknam S. Bakhoda M. (2024). Curcumin effects on glycaemic indices, lipid profile, blood pressure, inflammatory markers and anthropometric measurements of non-alcoholic fatty liver disease patients: a systematic review and meta-analysis of randomized clinical trials. Complement. Ther. Med. 80, 103025. 10.1016/j.ctim.2024.103025 38232906

[B16] Emami E. Heidari-Soureshjani S. Sherwin C. M. (2022). Anti-inflammatory response to curcumin supplementation in chronic kidney disease and hemodialysis patients: a systematic review and meta-analysis. Avicenna J. Phytomed 12 (6), 576–588. 10.22038/AJP.2022.20049 36583173 PMC9768855

[B17] Fan H.-Y. Wang X.-K. Li X. Ji K. Du S.-H. Liu Y. (2020). Curcumin, as a pleiotropic agent, improves doxorubicin-induced nephrotic syndrome in rats. J. Ethnopharmacol. 250, 112502. 10.1016/j.jep.2019.112502 31881321

[B18] Fathi S. Agharloo S. Falahatzadeh M. Bahraminavid S. Homayooni A. Faghfouri A. H. (2024). Effect of curcumin supplementation on symptoms of anxiety: a systematic review and meta-analysis of randomized controlled trials. Clin. Nutr. ESPEN 62, 253–259. 10.1016/j.clnesp.2024.05.017 38857152

[B19] Gomez-Pinilla F. Nguyen T. T. J. (2012). Natural mood foods: the actions of polyphenols against psychiatric and cognitive disorders. Nutr. Neurosci. 15 (3), 127–133. 10.1179/1476830511Y.0000000035 22334236 PMC3355196

[B20] Gouda M. M. Balaya R. D. A. Modi P. K. Kadri S. Chanderasekaran J. Balnadupete A. (2024). Impact of curcumin on the IL-17a-mediated p53-fibrinolytic system: mouse proteomics and integrated human fibrosis scRNAseq insights. Inflammation. 10.1007/s10753-024-02167-3 39424752

[B21] Gu Y. Niu Q. Zhang Q. Zhao Y. (2024). Ameliorative effects of curcumin on type 2 diabetes mellitus. Molecules 29 (12), 2934. 10.3390/molecules29122934 38930998 PMC11206386

[B22] Guyatt G. Oxman A. D. Akl E. A. Kunz R. Vist G. Brozek J. (2011). GRADE guidelines: 1. Introduction-GRADE evidence profiles and summary of findings tables. J. Clin. Epidemiol. 64 (4), 383–394. 10.1016/j.jclinepi.2010.04.026 21195583

[B23] Hayashino Y. Noguchi Y. Fukui T. (2005). Systematic evaluation and comparison of statistical tests for publication bias. J. Epidemiol. 15 (6), 235–243. 10.2188/jea.15.235 16276033 PMC7904376

[B24] Jamali N. Adib-Hajbaghery M. Soleimani A. (2020). The effect of curcumin ointment on knee pain in older adults with osteoarthritis: a randomized placebo trial. BMC Complement. Med. Ther. 20 (1), 305. 10.1186/s12906-020-03105-0 33032585 PMC7545864

[B25] Jena A. B. Kanungo N. Nayak V. Chainy G. B. N. Dandapat J. (2021). Catechin and curcumin interact with S protein of SARS-CoV2 and ACE2 of human cell membrane: insights from computational studies. Sci. Rep. 11 (1), 2043. 10.1038/s41598-021-81462-7 33479401 PMC7820253

[B26] Jiménez-Flores L. M. López-Briones S. Macías-Cervantes M. H. Ramírez-Emiliano J. Pérez-Vázquez V. (2014). A PPARγ, NF-κB and AMPK-dependent mechanism may be involved in the beneficial effects of curcumin in the diabetic db/db mice liver. Molecules 19 (6), 8289–8302. 10.3390/molecules19068289 24945581 PMC6271620

[B27] Kasprzak-Drozd K. Niziński P. Hawrył A. Gancarz M. Hawrył D. Oliwa W. (2024). Potential of curcumin in the management of skin diseases. Int. J. Mol. Sci. 25 (7), 3617. 10.3390/ijms25073617 38612433 PMC11012053

[B28] Kasprzak-Drozd K. Oniszczuk T. Gancarz M. Kondracka A. Rusinek R. Oniszczuk A. (2022). Curcumin and weight loss: does it work? Int. J. Mol. Sci. 23 (2), 639. 10.3390/ijms23020639 35054828 PMC8775659

[B29] Keller A. Wallace T. C. (2021). Tea intake and cardiovascular disease: an umbrella review. Ann. Med. 53 (1), 929–944. 10.1080/07853890.2021.1933164 34396859 PMC8366653

[B30] Khan S. U. Khan M. U. Riaz H. Valavoor S. Zhao D. Vaughan L. (2019). Effects of nutritional supplements and dietary interventions on cardiovascular outcomes: an umbrella review and evidence map. Ann. Intern Med. 171 (3), 190–198. 10.7326/M19-0341 31284304 PMC7261374

[B31] Kou H. Huang L. Jin M. He Q. Zhang R. Ma J. (2023). Effect of curcumin on rheumatoid arthritis: a systematic review and meta-analysis. Front. Immunol. 14, 1121655. 10.3389/fimmu.2023.1121655 37325651 PMC10264675

[B32] Li P. Ding L. Cao S. Feng X. Zhang Q. Chen Y. (2020). Curcumin metabolites contribute to the effect of curcumin on ameliorating insulin sensitivity in high-glucose-induced insulin-resistant HepG2 cells. J. Ethnopharmacol. 259, 113015. 10.1016/j.jep.2020.113015 32464315

[B33] Li Z. Wang Y. Xu Q. Ma J. Li X. Yan J. (2023). Berberine and health outcomes: an umbrella review. Phytother. Res. 37 (5), 2051–2066. 10.1002/ptr.7806 36999891

[B34] Liu X. Eyles J. McLachlan A. J. Mobasheri A. (2018). Which supplements can I recommend to my osteoarthritis patients? Rheumatol. Oxf. 57 (Suppl. l_4), iv75–iv87. 10.1093/rheumatology/key005 29506080

[B35] Malekmakan L. Hamidianjahromi A. Sayadi M. Rezazadeh M. H. (2022). Efficacy and safety of turmeric dietary supplementation on proteinuria in CKD: a systematic review and meta-analysis of RCT. Iran. J. Kidney Dis. 16 (3), 153–161. 10.52547/ijkd.6772 35714209

[B36] Maouche A. Boumediene K. Baugé C. (2024). Bioactive compounds in osteoarthritis: molecular mechanisms and therapeutic roles. Int. J. Mol. Sci. 25 (21), 11656. 10.3390/ijms252111656 39519204 PMC11546619

[B37] Mirzaei Dahka S. Afsharfar M. Tajaddod S. Sohouli M. H. Shekari S. Bakhshi Nafouti F. (2023). Impact of curcumin supplementation on radiation dermatitis severity: a systematic review and meta-analysis of randomized controlled trials. Asian Pac J. Cancer Prev. 24 (3), 783–789. 10.31557/APJCP.2023.24.3.783 36974529 PMC10334089

[B38] Mobasheri A. Spring-Charles A. Gamaleri F. C. McSwan J. Garg M. Sethi V. S. (2024). Evidence-based opinions from multidisciplinary experts on use of naturopathic herbal remedies in pain management. J. Pain Res. 17, 599–608. 10.2147/JPR.S432090 38347854 PMC10860847

[B39] Nakmareong S. Kukongviriyapan U. Pakdeechote P. Donpunha W. Kukongviriyapan V. Kongyingyoes B. (2011). Antioxidant and vascular protective effects of curcumin and tetrahydrocurcumin in rats with L-NAME-induced hypertension. Naunyn Schmiedeb. Arch. Pharmacol. 383 (5), 519–529. 10.1007/s00210-011-0624-z 21448566

[B40] Ng Q. X. Soh A. Y. S. Loke W. Venkatanarayanan N. Lim D. Y. Yeo W.-S. (2018). A meta-analysis of the clinical use of curcumin for irritable bowel syndrome (IBS). J. Clin. Med. 7 (10), 298. 10.3390/jcm7100298 30248988 PMC6210149

[B41] Nirgude S. Desai S. Ravindran F. Mhatre A. Mahadeva R. Sharma S. (2025). Global transcriptome profiling of ST09 treated breast cancer cells identifies miR-197-5p/GPX3 antioxidant axis as a regulator of tumorigenesis. Int. Immunopharmacol. 148, 114127. 10.1016/j.intimp.2025.114127 39870007

[B42] Page M. J. McKenzie J. E. Bossuyt P. M. Boutron I. Hoffmann T. C. Mulrow C. D. (2021). Updating guidance for reporting systematic reviews: development of the PRISMA 2020 statement. J. Clin. Epidemiol. 134, 103–112. 10.1016/j.jclinepi.2021.02.003 33577987

[B43] Panknin T. M. Howe C. L. Hauer M. Bucchireddigari B. Rossi A. M. Funk J. L. (2023). Curcumin supplementation and human disease: a scoping review of clinical trials. Int. J. Mol. Sci. 24 (5), 4476. 10.3390/ijms24054476 36901908 PMC10003109

[B44] Ramaswami G. Chai H. Yao Q. Lin P. H. Lumsden A. B. Chen C. (2004). Curcumin blocks homocysteine-induced endothelial dysfunction in porcine coronary arteries. J. Vasc. Surg. 40 (6), 1216–1222. 10.1016/j.jvs.2004.09.021 15622377

[B45] Rungseesantivanon S. Thenchaisri N. Ruangvejvorachai P. Patumraj S. (2010). Curcumin supplementation could improve diabetes-induced endothelial dysfunction associated with decreased vascular superoxide production and PKC inhibition. BMC Complement. Altern. Med. 10, 57. 10.1186/1472-6882-10-57 20946622 PMC2964550

[B46] Sadeghi N. Mansoori A. Shayesteh A. Hashemi S. J. (2020). The effect of curcumin supplementation on clinical outcomes and inflammatory markers in patients with ulcerative colitis. Phytother. Res. 34 (5), 1123–1133. 10.1002/ptr.6581 31802559

[B47] Sadeghian M. Rahmani S. Jamialahmadi T. Johnston T. P. Sahebkar A. (2021). The effect of oral curcumin supplementation on health-related quality of life: a systematic review and meta-analysis of randomized controlled trials. J. Affect Disord. 278, 627–636. 10.1016/j.jad.2020.09.091 33038707

[B48] Sahebkar A. Henrotin Y. (2016). Analgesic efficacy and safety of curcuminoids in clinical practice: a systematic review and meta-analysis of randomized controlled trials. Pain Med. 17 (6), 1192–1202. 10.1093/pm/pnv024 26814259

[B49] Sandoval-Ramírez B.-A. Catalán Ú. Llauradó E. Valls R.-M. Salamanca P. Rubió L. (2022). The health benefits of anthocyanins: an umbrella review of systematic reviews and meta-analyses of observational studies and controlled clinical trials. Nutr. Rev. 80 (6), 1515–1530. 10.1093/nutrit/nuab086 34725704

[B50] Sarraf P. Parohan M. Javanbakht M. H. Ranji-Burachaloo S. Djalali M. (2019). Short-term curcumin supplementation enhances serum brain-derived neurotrophic factor in adult men and women: a systematic review and dose-response meta-analysis of randomized controlled trials. Nutr. Res. 69, 1–8. 10.1016/j.nutres.2019.05.001 31279955

[B51] Shafiee A. Athar M. M. T. Shahid A. Ghafoor M. S. Ayyan M. Zahid A. (2023). Curcumin for the treatment of COVID-19 patients: a meta-analysis of randomized controlled trials. Phytother. Res. 37 (3), 1167–1175. 10.1002/ptr.7724 36640146

[B52] Shao W. Yu Z. Chiang Y. Yang Y. Chai T. Foltz W. (2012). Curcumin prevents high fat diet induced insulin resistance and obesity via attenuating lipogenesis in liver and inflammatory pathway in adipocytes. PLoS One 7 (1), e28784. 10.1371/journal.pone.0028784 22253696 PMC3253779

[B53] Sharifipour F. Siahkal S. F. Qaderi K. Mohaghegh Z. Zahedian M. Azizi F. (2024). Effect of curcumin on dysmenorrhea and symptoms of premenstrual syndrome: a systematic review and meta-analysis. Korean J. Fam. Med. 45 (2), 96–104. 10.4082/kjfm.23.0184 38266637 PMC10973707

[B54] Shea B. J. Reeves B. C. Wells G. Thuku M. Hamel C. Moran J. (2017). AMSTAR 2: a critical appraisal tool for systematic reviews that include randomised or non-randomised studies of healthcare interventions, or both. BMJ 358, j4008. 10.1136/bmj.j4008 28935701 PMC5833365

[B55] Shen W. Qu Y. Jiang H. Wang H. Pan Y. Zhang Y. (2022). Therapeutic effect and safety of curcumin in women with PCOS: a systematic review and meta-analysis. Front. Endocrinol. (Lausanne) 13, 1051111. 10.3389/fendo.2022.1051111 36387924 PMC9646792

[B56] Sideri S. Papageorgiou S. N. Eliades T. (2018). Registration in the international prospective register of systematic reviews (PROSPERO) of systematic review protocols was associated with increased review quality. J. Clin. Epidemiol. 100, 103–110. 10.1016/j.jclinepi.2018.01.003 29339215

[B57] Spanoudaki M. Papadopoulou S. K. Antasouras G. Papadopoulos K. A. Psara E. Vorvolakos T. (2024). Curcumin as a multifunctional spice ingredient against mental disorders in humans: current clinical studies and bioavailability concerns. Life (Basel) 14 (4), 479. 10.3390/life14040479 38672750 PMC11050944

[B58] Suphim B. Prawan A. Kukongviriyapan U. Kongpetch S. Buranrat B. Kukongviriyapan V. (2010). Redox modulation and human bile duct cancer inhibition by curcumin. Food Chem. Toxicol. 48 (8-9), 2265–2272. 10.1016/j.fct.2010.05.059 20510329

[B59] Tian J. Feng B. Tian Z. (2022). The effect of curcumin on lipid profile and glycemic status of patients with type 2 diabetes mellitus: a systematic review and meta-analysis. Evid. Based Complement. Altern. Med. 2022, 8278744. 10.1155/2022/8278744 PMC9232354 35754684

[B60] Wang J. Ghosh S. S. Ghosh S. (2017). Curcumin improves intestinal barrier function: modulation of intracellular signaling, and organization of tight junctions. Am. J. Physiol. Cell Physiol. 312 (4), C438–C445. 10.1152/ajpcell.00235.2016 28249988 PMC5407015

[B61] Wang Y. Wang S. Ma C. Qi W. Lv J. Zhang M. (2024a). Nrf2 depletion enhanced curcumin therapy effect in gastric cancer by inducing the excessive accumulation of ROS. Sci. Rep. 14 (1), 30165. 10.1038/s41598-024-81375-1 39627516 PMC11615379

[B62] Wang Y. Zhou D. Zhang X. Qing M. Li X. Chou Y. (2024b). Curcumin promotes renewal of intestinal epithelium by miR-195-3p. J. Ethnopharmacol. 320, 117413. 10.1016/j.jep.2023.117413 37972911

[B63] Wang Z. Singh A. Jones G. Winzenberg T. Ding C. Chopra A. (2021a). Efficacy and safety of turmeric extracts for the treatment of knee osteoarthritis: a systematic review and meta-analysis of randomised controlled trials. Curr. Rheumatol. Rep. 23 (2), 11. 10.1007/s11926-020-00975-8 33511486

[B64] Wang Z. Zhang Q. Huang H. Liu Z. (2021b). The efficacy and acceptability of curcumin for the treatment of depression or depressive symptoms: a systematic review and meta-analysis. J. Affect Disord. 282, 242–251. 10.1016/j.jad.2020.12.158 33418373

[B65] Weissgerber T. L. (2021). Training early career researchers to use meta-research to improve science: a participant-guided “learn by doing” approach. PLoS Biol. 19 (2), e3001073. 10.1371/journal.pbio.3001073 33626038 PMC7904195

[B66] Yeung A. W. K. Horbańczuk M. Tzvetkov N. T. Mocan A. Carradori S. Maggi F. (2019). Curcumin: total-scale analysis of the scientific literature. Molecules 24 (7), 1393. 10.3390/molecules24071393 30970601 PMC6480685

[B67] Yin J. Wei L. Wang N. Li X. Miao M. (2022). Efficacy and safety of adjuvant curcumin therapy in ulcerative colitis: a systematic review and meta-analysis. J. Ethnopharmacol. 289, 115041. 10.1016/j.jep.2022.115041 35091013

[B68] Yu Y. Wu S. Li J. Wang R. Xie X. Yu X. (2015). The effect of curcumin on the brain-gut axis in rat model of irritable bowel syndrome: involvement of 5-HT-dependent signaling. Metab. Brain Dis. 30 (1), 47–55. 10.1007/s11011-014-9554-z 24807589

[B69] Zeng L. Yu G. Hao W. Yang K. Chen H. (2021). The efficacy and safety of Curcuma longa extract and curcumin supplements on osteoarthritis: a systematic review and meta-analysis. Biosci. Rep. 41 (6). 10.1042/BSR20210817 PMC8202067 34017975

[B70] Zhang J. Huang Y. Xu J. Zhao R. Xiong C. Habu J. (2022). Global publication trends and research hotspots of curcumin application in tumor: a 20-year bibliometric approach. Front. Oncol. 12, 1033683. 10.3389/fonc.2022.1033683 36300100 PMC9589263

[B71] Zhang M.-W. Sun X. Xu Y.-W. Meng W. Tang Q. Gao H. (2024). Curcumin relieves oxaliplatin-induced neuropathic pain via reducing inflammation and activating antioxidant response. Cell Biol. Int. 48 (6), 872–882. 10.1002/cbin.12153 38480956

[B72] Zhang X. Zhang H. Wang J. Chen Y. Lin J. Wang Q. (2025). Curcumin attenuates ulcerative colitis via regulation of Sphingosine kinases 1/NF-κB signaling pathway. Biofactors 51 (1), e70001. 10.1002/biof.70001 39832759

[B73] Zhu L.-N. Mei X. Zhang Z.-G. Xie Y.-P. Lang F. (2019). Curcumin intervention for cognitive function in different types of people: a systematic review and meta-analysis. Phytother. Res. 33 (3), 524–533. 10.1002/ptr.6257 30575152

