# Supplementary material for: Curcumin and multiple health outcomes: critical umbrella review of intervention meta-analyses
Source: Front Pharmacol. 2025 Jun 5;16:1601204. doi: 10.3389/fphar.2025.1601204 (PMC12176752; doi:10.3389/fphar.2025.1601204)
Supplement: Supplementary file 1 [file Table1.docx]

Supplementary Material

Supplementary Table 1. Electronic Database Search Strategies

Searches executed 18 Jun 2024; total number records retrieved: 1628

| **Pubmed**  464 results  ((("curcumin"[MeSH Terms]) OR ((((((curcumin[Title/Abstract]) OR (curcuma[Title/Abstract])) OR (turmeric[Title/Abstract])) OR (curcuminoids[Title/Abstract])) OR (curcuminoid[Title/Abstract])) OR (Diferuloylmethane[Title/Abstract])) OR (curcumae Longae Rhizoma[Title/Abstract]))) AND ((("Meta-Analysis as Topic"[MeSH Terms]) OR ("Systematic Reviews as Topic"[MeSH Terms])) OR ((meta analysis[Title/Abstract]) OR (systematic review[Title/Abstract]))) |
| --- |
| **Embase**  1157 results  ((('curcumin'/exp) OR (curcumin:ti,ab,kw) OR (curcuma:ti,ab,kw) OR (turmeric:ti,ab,kw) OR (curcuminoids:ti,ab,kw) OR (curcuminoid:ti,ab,kw) OR (diferuloylmethane:ti,ab,kw) OR ('curcumae longae rhizoma':ti,ab,kw) )AND (('meta analysis'/exp) OR ('systematic review'/exp) OR ('systematic review':ti,ab,kw) OR ('meta analysis':ti,ab,kw))) AND [humans]/lim NOT ([conference abstract]/lim OR [editorial]/lim OR [letter]/lim OR [short survey]/lim) |
| **Cochrane**  7 results  ((MeSH descriptor: [Curcumin] explode all trees) OR ((curcumin):ti,ab,kw OR (curcuma):ti,ab,kw OR (turmeric):ti,ab,kw OR (curcuminoids):ti,ab,kw OR (curcuminoid):ti,ab,kw OR (Diferuloylmethane):ti,ab,kw OR (curcumae Longae Rhizoma ):ti,ab,kw)) Limited to Cochrane Reviews |
