# Supplementary material for: Curcumin and multiple health outcomes: critical umbrella review of intervention meta-analyses
Source: Front Pharmacol. 2025 Jun 5;16:1601204. doi: 10.3389/fphar.2025.1601204 (PMC12176752; doi:10.3389/fphar.2025.1601204)
Supplement: Supplementary file 2 [file Table2.docx]

Supplementary Material

**Supplementary Table S2 GRADE profile of Curcumin on health outcomes**

^a^ The included study had an unclear risk of selection, performance, detection, and reporting biases; ^b^ 50% ≤ I^2^ < 75%; ^c^ I^2^ ≥ 75%; ^d^ 95 % Cl includes invalid line; ^e^ Sample size <300; ^f^ Funnel plot or Egger's or Begg's tests indicated asymmetry.

| **Reference** | **Outcomes** | **Population** | **Interventions/ comparators** | **Number of RCTs** | **Sample size** | **Effect metrics** | **Estimates** | **95%CI** | **I^2^** | **P value** | **Risk of bias** | **Inconsistency** | **Indirection** | **Imprecision** | **publication bias** | **GRADE level** |
| --- | --- | --- | --- | --- | --- | --- | --- | --- | --- | --- | --- | --- | --- | --- | --- | --- |
| **Glucose metabolic and insulin secretion** | | | | | | | | | | | | | | | | |
| Tian(Tian et al., 2022) | FBG | T2DM | Curcumin/placebo, medication | 9 | NR | Random/WMD | -8.85 | -14.4, -3.29 | 41.2% | 0.002 | Serious ^a^ | Not serious | Not serious | Not serious | Not serious | **⨁⨁⨁◯**  **Moderate** |
| Ebrahimzadeh(Ebrahimzadeh et al., 2024) | FBG | NAFLD | Curcumin/placebo | 12 | NR | Random/WMD | -2.83 | -4.61, -1.06 | 51.3% | NA | Serious ^a^ | Serious ^b^ | Not serious | Not serious | NR | **⨁⨁◯◯**  **Low** |
| Shen(Shen et al., 2022) | FBG | PCOS | Curcumin/placebo, medication | 7 | 447(NR) | Random/WMD | -3.618 | -5.165, -2.071 | 20.4% | <0.001 | Serious ^a^ | Not serious | Not serious | Not serious | NR | **⨁⨁⨁◯**  **Moderate** |
| Tian(Tian et al., 2022) | HbA1c | T2DM | Curcumin/placebo, medication | 8 | NR | Random/WMD | -0.54 | -0.81, -0.27 | 65.2% | ≤0.001 | Serious ^a^ | Serious ^b^ | Not serious | Not serious | Not serious | **⨁⨁◯◯**  **Low** |
| Ebrahimzadeh(Ebrahimzadeh et al., 2024) | HbA1c | NAFLD | Curcumin/placebo | 4 | NR | Random/WMD | -0.17 | -0.44, 0.11 | 92.4% | NA | Serious ^a^ | Very serious ^c^ | Not serious | Very serious ^d, e^ | NR | **⨁◯◯◯**  **Very low** |
| Shen(Shen et al., 2022) | HbA1c | PCOS | Curcumin/placebo, medication | 2 | NR | Random/WMD | -0.042 | -0.471, 0.387 | 56.8% | 0.849 | Serious ^a^ | Not serious | Not serious | Serious ^d^ | NR | **⨁⨁◯◯**  **Low** |
| Ebrahimzadeh(Ebrahimzadeh et al., 2024) | Insulin | NAFLD | Curcumin/placebo | 4 | NR | Random/WMD | -0.14 | -1.03, 0.76 | 83.0% | NA | Serious ^a^ | Very serious ^c^ | Not serious | Very serious ^d, e^ | NR | **⨁◯◯◯**  **Very low** |
| Shen(Shen et al., 2022) | Insulin | PCOS | Curcumin/placebo, medication | 7 | NR | Random/WMD | -1.834 | -2.701, -0.968 | 8.4% | <0.001 | Serious ^a^ | Not serious | Not serious | Not serious | NR | **⨁⨁⨁◯**  **Moderate** |
| Ebrahimzadeh(Ebrahimzadeh et al., 2024) | HOMA-IR | NAFLD | Curcumin/placebo | 4 | NR | Random/WMD | -0.52 | -0.84, -0.20 | 82.8% | NA | Serious ^a^ | Very serious ^c^ | Not serious | Serious ^e^ | NR | **⨁◯◯◯**  **Very low** |
| Shen(Shen et al., 2022) | HOMA-IR | PCOS | Curcumin/placebo, medication | 7 | 447(NR) | Random/WMD | -0.565 | -0.779, -0.351 | 0% | <0.001 | Serious ^a^ | Not serious | Not serious | Not serious | NR | **⨁⨁⨁◯**  **Moderate** |
| Ebrahimzadeh(Ebrahimzadeh et al., 2024) | QUICKI | NAFLD | Curcumin/placebo | 4 | NR | Random/WMD | 0.01 | -0.00, 0.01 | 96.2% | NA | Serious ^a^ | Very serious ^c^ | Not serious | Very serious ^d, e^ | NR | **⨁◯◯◯**  **Very low** |
| Shen(Shen et al., 2022) | QUICKI | PCOS | Curcumin/placebo, medication | 4 | NR | Random/WMD | 0.011 | 0.005, 0.017 | 39.6% | <0.001 | Serious ^a^ | Not serious | Not serious | Serious ^e^ | NR | **⨁⨁◯◯**  **Low** |
| Shen(Shen et al., 2022) | Glu120 | PCOS | Curcumin/placebo, medication | 2 | NR | Random/WMD | -0.063 | -2.307, 2.181 | 87.4% | 0.956 | Serious ^a^ | Very serious ^c^ | Not serious | Very serious ^d,^ ^e^ | NR | **⨁◯◯◯**  **Very low** |
| Shen(Shen et al., 2022) | Ins120 | PCOS | Curcumin/placebo, medication | 2 | NR | Random/WMD | -12.445 | -44.384, 19.494 | 0.0% | 0.445 | Serious ^a^ | Not serious | Not serious | Very serious ^d,^ ^e^ | NR | **⨁◯◯◯**  **Very low** |
| **Blood pressure and endothelial function** | | | | | | | | | | | | | | | | |
| Dehzad(Dehzad et al., 2024) | SBP | Adults | Curcumin/turmeric supplementation/ placebo | 29 | NR | Random/WMD | -2.02 | -2.85, -1.18 | 96.7% | 0.000 | Serious ^a^ | Very serious ^c^ | Not serious | Not serious | NR | **⨁◯◯◯**  **Very low** |
| Ebrahimzadeh(Ebrahimzadeh et al., 2024) | SBP | NAFLD | Curcumin/ placebo | 5 | NR | Random/WMD | -0.93 | -2.36, 0.50 | 83.4% | NA | Serious ^a^ | Very serious ^c^ | Not serious | Very serious ^d, e^ | NR | **⨁◯◯◯**  **Very low** |
| Dehzad(Dehzad et al., 2024) | DBP | Adults | Curcumin/turmeric supplementation/ placebo | 27 | NR | Random/WMD | 0.82 | -1.46, -0.18 | 93.2% | 0.00 | Serious ^a^ | Very serious ^c^ | Not serious | Not serious | NR | **⨁◯◯◯**  **Very low** |
| Ebrahimzadeh(Ebrahimzadeh et al., 2024) | DBP | NAFLD | Curcumin/placebo | 5 | NR | Random/WMD | -1.37 | -3.09, 0.35 | 90.5% | NA | Serious ^a^ | Very serious ^c^ | Not serious | Very serious ^d, e^ | NR | **⨁◯◯◯**  **Very low** |
| Dehzad(Dehzad et al., 2024) | VCAM-1 | Adults | Curcumin/turmeric supplementation/ placebo | 4 | NR | Random/WMD | -39.19 | -66.15, -12.23 | 73% |  | Serious ^a^ | Serious ^b^ | Not serious | Not serious | NR | **⨁⨁◯◯**  **Low** |
| Dehzad(Dehzad et al., 2024) | FMD | Adults | Curcumin/turmeric supplementation/ placebo | 3 | NR | Random/WMD | 2.00% | 1.07, 2.94 | 79.5% | 0.002 | Serious ^a^ | Very serious ^c^ | Not serious | Not serious | NR | **⨁◯◯◯**  **Very low** |
| Dehzad(Dehzad et al., 2024) | ICAM-1 | Adults | Curcumin/turmeric supplementation/ placebo | 3 | NR | Random/WMD | -17.05 | -80.79, 46.70 | 94.1% | 0.000 | Serious ^a^ | Very serious ^c^ | Not serious | Serious ^d^ | NR | **⨁◯◯◯**  **Very low** |
| Dehzad(Dehzad et al., 2024) | PWV | Adults | Curcumin/turmeric supplementation/ placebo | 6 | NR | Random/WMD | -79.53 | -210.38, 51.33 | 99.7% | 0.000 | Serious ^a^ | Very serious ^c^ | Not serious | Serious ^d^ | NR | **⨁◯◯◯**  **Very low** |
| **Lipid profile** | | | | | | | | | | | | | | | | |
| Dehzad(Dehzad et al., 2023a) | TC | Adults | Curcumin/turmeric supplementation/placebo | 55 | NR | Random/WMD | -3.99 | -5.33, -2.65 | 97.0% | NR | Serious ^a^ | Very serious ^c^ | Not serious | Not serious | Not serious | **⨁◯◯◯**  **Very low** |
| Shen(Shen et al., 2022) | TC | PCOS | Curcumin/placebo, medication | 5 | NR | Random/WMD | -15.591 | -27.908, -3.273 | 68.9% | 0.013 | Serious ^a^ | Serious ^b^ | Not serious | Serious ^e^ | NR | **⨁◯◯◯**  **Very low** |
| Tian(Tian et al., 2022) | TC | T2DM | Curcumin/placebo, medication | 9 | NR | Random/WMD | -8.91 | -14.18, -3.63 | 28.9% | 0.001 | Serious ^a^ | Not serious | Not serious | Not serious | Not serious | **⨁⨁⨁◯**  **Moderate** |
| Dehzad(Dehzad et al., 2023a) | TG | Adults | Curcumin/turmeric supplementation/placebo | 58 | NR | Random/WMD | -6.69 | -7.93, -5.45 | 95.7% | NR | Serious ^a^ | Very serious ^c^ | Not serious | Not serious | Not serious | **⨁◯◯◯**  **Very low** |
| Shen(Shen et al., 2022) | TG | PCOS | Curcumin/placebo, medication | 5 | NR | Random/WMD | -8.889 | -27.246 ，9.468 | 91.5% | 0.343 | Serious ^a^ | Very serious ^c^ | Not serious | Serious ^d^ | NR | **⨁◯◯◯**  **Very low** |
| Tian(Tian et al., 2022) | TG | T2DM | Curcumin/placebo, medication | 9 | NR | Random/WMD | - 18.97 | -36.47,1.47 | 80.5% | 0.03 | Serious ^a^ | Very serious ^c^ | Not serious | Serious ^d^ | Not serious | **⨁◯◯◯**  **Very low** |
| Dehzad(Dehzad et al., 2023a) | LDL-C | Adults | Curcumin/turmeric supplementation/placebo | 57 | NR | Random/WMD | -4.89 | -5.92, -3.87 | 95.6% | NR | Serious ^a^ | Very serious ^c^ | Not serious | Not serious | Not serious | **⨁◯◯◯**  **Very low** |
| Tian(Tian et al., 2022) | LDL-C | T2DM | Curcumin/placebo, medication | 9 | NR | Random/WMD | -4.01 | -10.96, 2.95 | 49.7% | 0.259 | Serious ^a^ | Not serious | Not serious | Serious ^d^ | Not serious | **⨁⨁◯◯**  **Low** |
| Shen(Shen et al., 2022) | LDL-C | PCOS | Curcumin/placebo, medication | 5 | NR | Random/WMD | -6.427 | -17.343, 4.489 | 78.8% | 0.249 | Serious ^a^ | Very serious ^c^ | Not serious | Serious ^d^ | NR | **⨁◯◯◯**  **Very low** |
| Dehzad(Dehzad et al., 2023a) | HDL-C | Adults | Curcumin/turmeric supplementation/placebo | 59 | NR | Random/WMD | 1.80 | 1.43, 2.17 | 95.0% | NR | Serious ^a^ | Very serious ^c^ | Not serious | Not serious | Not serious | **⨁◯◯◯**  **Very low** |
| Shen(Shen et al., 2022) | HDL-C | PCOS | Curcumin/placebo, medication | 5 | NR | Random/WMD | 3.713 | -0.786, 8.211 | 81.3% | 0.106 | Serious ^a^ | Very serious ^c^ | Not serious | Serious ^d^ | NR | **⨁◯◯◯**  **Very low** |
| Tian(Tian et al., 2022) | HDL-C | T2DM | Curcumin/placebo, medication | 9 | NR | Random/WMD | 0.32 | -0.74, 1.37 | 19.1% | 0.557 | Serious ^a^ | Not serious | Not serious | Serious ^d^ | Not serious | **⨁⨁◯◯**  **Low** |
| Dehzad(Dehzad et al., 2023a) | Apo-A | Adults | Curcumin/turmeric supplementation/placebo | 2 | NR | Random/WMD | 1.58 | -3.49, 6.56 | 64.4% | NR | Serious ^a^ | Serious ^b^ | Not serious | Serious ^d^ | Not serious | **⨁◯◯◯**  **Very low** |
| Dehzad(Dehzad et al., 2023a) | Apo-B | Adults | Curcumin/turmeric supplementation/placebo | 2 | NR | Random/WMD | 1.35 | -9.74, 12.44 | 83.4% | NR | Serious ^a^ | Very serious ^c^ | Not serious | Serious ^d^ | Not serious | **⨁◯◯◯**  **Very low** |
| Anthropometric parameters | | | | | | | | | | | | | | | | |
| Shen(Shen et al., 2022) | Body weight | PCOS | Curcumin/placebo, medication | 4 | NR | Random/WMD | -0.924, | -2.009, 0.162, | 45.2% | 0.095 | Serious ^a^ | Not serious | Not serious | Very serious ^d, e^ | NR | **⨁◯◯◯**  **Very low** |
| Shen(Shen et al., 2022) | WC | PCOS | Curcumin/placebo | 2 | NR | Random/WMD | -1.475 | -4.519, 1.570 | 81.6% | 0.342 | Serious ^a^ | Very serious ^c^ | Not serious | Very serious ^d, e^ | NR | **⨁◯◯◯**  **Very low** |
| Shen(Shen et al., 2022) | BMI | PCOS | Curcumin/placebo, medication | 7 | 447(225/222) | Random/WMD | -0.267 | -0.450, -0.084 | 0% | 0.004 | Serious ^a^ | Not serious | Not serious | Serious ^d^ | NR | **⨁⨁◯◯**  **Low** |
| Ebrahimzadeh(Ebrahimzadeh et al., 2024) | BMI | NAFLD | Curcumin/placebo | 16 | NR | Random/WMD | -0.35 | -0.57, -0.13 | 0.0% | NA | Serious ^a^ | Not serious | Not serious | Not serious | NR | **⨁⨁⨁◯**  **Moderate** |
| Shen(Shen et al., 2022) | WHR | PCOS | Curcumin/placebo, medication | 2 | NR | Random/WMD | -0.024 | -0.048, 0.000 | 0% | 0.052 | Serious ^a^ | Not serious | Not serious | Very serious ^d, e^ | NR | **⨁◯◯◯**  **Very low** |
| Dehzad(Dehzad et al., 2023d) | BW | Adults | Curcumin/placebo | 38 | NR | Random/WMD | -0.82 | -1.30, -0.35 | 78.7% | 0.001 | Serious ^a^ | Very serious ^c^ | Not serious | Not serious | Not serious | **⨁◯◯◯**  **Very low** |
| Ebrahimzadeh(Ebrahimzadeh et al., 2024) | BW | NAFLD | Curcumin/placebo | 13 | NR | Random/WMD | -0.81 | -1.28, -0.35 | 0.0% | NA | Serious ^a^ | Not serious | Not serious | Not serious | NR | **⨁⨁⨁◯**  **Moderate** |
| Dehzad(Dehzad et al., 2023d) | BMI | Adults | Curcumin/placebo | 45 | NR | Random/WMD | -0.30 | -0.53, -0.06 | 94.7% | 0.013 | Serious ^a^ | Very serious ^c^ | Not serious | Not serious | Not serious | **⨁◯◯◯**  **Very low** |
| Dehzad(Dehzad et al., 2023d) | WC | Adults | Curcumin/placebo | 22 | NR | Random/WMD | -1.31 | -1.94, -0.69 | 78.5% | <0.001 | Serious ^a^ | Very serious ^c^ | Not serious | Not serious | Not serious | **⨁◯◯◯**  **Very low** |
| Ebrahimzadeh(Ebrahimzadeh et al., 2024) | WC | NAFLD | Curcumin/placebo | 7 | NR | Random/WMD | -01.80 | -3.61, 0.02 | 87.2% | NA | Serious ^a^ | Very serious ^c^ | Not serious | Serious ^d^ | NR | **⨁◯◯◯**  **Very low** |
| Dehzad(Dehzad et al., 2023d) | BFP | Adults | Curcumin/placebo | 16 | NR | Random/WMD | -0.88 | -1.51, -0.25 | 86.2% | 0.007 | Serious ^a^ | Very serious ^c^ | Not serious | Not serious | Not serious | **⨁◯◯◯**  **Very low** |
| Dehzad(Dehzad et al., 2023d) | Leptin | Adults | Curcumin/placebo | 9 | NR | Random/WMD | -4.46 | -6.70, -2.21 | 96.1% | <0.001 | Serious ^a^ | Very serious ^c^ | Not serious | Not serious | Serious ^f^ | **⨁◯◯◯**  **Very low** |
| Dehzad(Dehzad et al., 2023d) | Adiponectin | Adults | Curcumin/placebo | 11 | NR | Random/WMD | 2.48 | 1.34, 3.62 | 96.3% | <0.001 | Serious ^a^ | Very serious ^c^ | Not serious | Not serious | Serious ^f^ | **⨁◯◯◯**  **Very low** |
| **Inflammatory and oxidative stress markers** | | | | | | | | | | | | | | | | |
| Dehzad(Dehzad et al., 2023c) | CRP | Adults | Curcumin/turmeric supplementation /placebo | 45 | NR | Random/WMD | -0.58 | -0.74, -0.41 | 98.9% | <0.001 | Serious ^a^ | Very serious ^c^ | Not serious | Not serious | Not serious | **⨁◯◯◯**  **Very low** |
| Shen(Shen et al., 2022) | CRP | PCOS | Curcumin/placebo | 2 | 81(42/39) | Random/WMD | -0.785 | -1.553, -0.017 | 23.9% | 0.252 | Serious ^a^ | Not serious | Not serious | Serious ^e^ | NR | **⨁⨁◯◯**  **Low** |
| Kou(Kou et al., 2023) | CRP | RA | Curcumin/placebo or standard of care | 6 | 223(113/110) | Random/MD | -0.93 | -1.33, -0.53 | 89% | <0.000001 | Serious ^a^ | Very serious ^c^ | Not serious | Serious ^e^ | Not serious | **⨁◯◯◯**  **Very low** |
| Ebrahimzadeh(Ebrahimzadeh et al., 2024) | CRP | NAFLD | Curcumin/placebo | 4 | NR | Random/WMD | -2.59 | -5.45, 0.26 | 99.4% |  | Serious ^a^ | Very serious ^c^ | Not serious | Very serious ^d, e^ | NR | **⨁◯◯◯**  **Very low** |
| Emami(Emami et al., 2022) | hs-CRP | CKD | Curcumin/placebo | 8 | 429 (214/215) | Random/SMD | -0.17 | -0.36, 0.03 | 79.6% | 0.093 | Serious ^a^ | Very serious ^c^ | Not serious | Serious ^d^ | Not serious | **⨁◯◯◯**  **Very low** |
| Kou(Kou et al., 2023) | ESR | RA | Curcumin/placebo or standard of care | 8 | 236(119/117) | Random/MD | -29.47 | -54.05, -4.88 | 99% | 0.02 | Serious ^a^ | Very serious ^c^ | Not serious | Serious ^e^ | Not serious | **⨁◯◯◯**  **Very low** |
| Dehzad(Dehzad et al., 2023c) | TNF-α | Adults | Curcumin/turmeric supplementation /placebo | 24 | NR | Random/WMD | -3.48 | -4.38, -2.58 | 99.4% | <0.001 | Serious ^a^ | Very serious ^c^ | Not serious | Not serious | Not serious | **⨁◯◯◯**  **Very low** |
| Beba(Beba et al., 2022) | TNF-α | Adults | Curcumin/placebo |  | NR | Random/WMD | -0.22 | -0.33, -0.10 | 93.2% |  | Not serious | Very serious ^c^ | Not serious | Serious ^e^ | Serious ^f^ | **⨁◯◯◯**  **Very low** |
| Emami(Emami et al., 2022) | TNF-α | CKD | Curcumin/placebo | 4 | 210(106/104) | Random/SMD | 0.11 | -0.19, 0.40 | 95.9% | 0.48 | Serious ^a^ | Very serious ^c^ | Not serious | Very serious ^d, e^ | Not serious | **⨁◯◯◯**  **Very low** |
| Ebrahimzadeh(Ebrahimzadeh et al., 2024) | TNF-α | NAFLD | Curcumin/placebo | 4 | NR | Random/WMD | -2.58 | -6.21, 1.06 | 98.6% |  | Serious ^a^ | Very serious ^c^ | Not serious | Very serious ^d, e^ | NR | **⨁◯◯◯**  **Very low** |
| Dehzad(Dehzad et al., 2023c) | IL-6 | Adults | Curcumin/turmeric supplementation /placebo | 20 | NR | Random/WMD | -1.31 | -1.58, -0.67 | 88.2% | <0.001 | Serious ^a^ | Very serious ^c^ | Not serious |  | Not serious | **⨁◯◯◯**  **Very low** |
| Emami(Emami et al., 2022) | IL-6 | CKD | Curcumin/placebo | 3 | 141(71/70) | Random/SMD | 0.24 | -0.14, 0.62 | 97.1% | 0.221 | Serious ^a^ | Very serious ^c^ | Not serious | Very serious ^d, e^ | Not serious | **⨁◯◯◯**  **Very low** |
| Beba(Beba et al., 2022) | IL-6 | Adults | Curcumin/placebo |  | NR |  | -0.05 | -0.14, 0.04 | 46.9% |  | Not serious | Not serious | Not serious | Very serious ^d, e^ | Not serious | **⨁⨁◯◯**  **Low** |
| Ebrahimzadeh(Ebrahimzadeh et al., 2024) | IL-6 | NAFLD | Curcumin/placebo | 3 | NR | Random/WMD | -1.67 | -3.80, 0.47 | 81.3% |  | Serious ^a^ | Very serious ^c^ | Not serious | Very serious ^d, e^ | NR | **⨁◯◯◯**  **Very low** |
| Beba(Beba et al., 2022) | IL-8 | Adults | Curcumin/placebo |  | NR |  | -0.33 | -1.39,0.73 | 85.6% |  | Not serious | Very serious ^c^ | Not serious | Very serious ^d, e^ | Not serious | **⨁◯◯◯**  **Very low** |
| Dehzad(Dehzad et al., 2023c) | IL-1β | Adults | Curcumin/turmeric supplementation /placebo | 6 | NR | Random/WMD | -0.46 | -1.18, 0.27 | 75.8 | 0.218 | Serious ^a^ | Very serious ^c^ | Not serious | Serious ^d^ | Serious ^f^ | **⨁◯◯◯**  **Very low** |
| Dehzad(Dehzad et al., 2023c) | TAC | Adults | Curcumin/turmeric supplementation /placebo | 16 | NR | Random/WMD | 0.21 | 0.08, 0.33 | 99.6% | 0.001 | Serious ^a^ | Very serious ^c^ | Not serious | Not serious | Not serious | **⨁◯◯◯**  **Very low** |
| Dehzad(Dehzad et al., 2023c) | MDA | Adults | Curcumin/turmeric supplementation /placebo | 18 | NR | Random/WMD | -0.33 | -0.53, -0.12 | 99.6% | 0.001 | Serious ^a^ | Very serious ^c^ | Not serious | Not serious | Not serious | **⨁◯◯◯**  **Very low** |
| Dehzad(Dehzad et al., 2023c) | SOD | Adults | Curcumin/turmeric supplementation /placebo | 7 | NR | Random/WMD | 20.51 | 7.35, 33.67 | 95.4% | 0.002 | Serious ^a^ | Very serious ^c^ | Not serious | Not serious | Not serious | **⨁◯◯◯**  **Very low** |
| **Gastrointestinal effects** | | | | | | | | | | | | | | | | |
| **Xiang Ng(Ng et al., 2018)** | IBS severity rating | IBS | Curcumin/placebo | 3 | 326 (NR） | Random/SMD | -0.466 | -1.113，0.182 | 85.22% | 0.158 | Serious ^a^ | Very serious ^c^ | Not serious | Serious ^d^ | NR | **⨁◯◯◯**  **Very low** |
| Yin(Yin et al., 2022) | Clinical remission | UC | Curcumin/placebo | 6 | 385(192/193) | Random/RR | 2.1 | 1.13, 3.89, | 80% | 0.02 | Not serious | Very serious ^c^ | Not serious | Not serious | NR | **⨁⨁◯◯**  **Low** |
| Yin(Yin et al., 2022) | Endoscopic remission | UC | Curcumin/placebo | 3 | 181(89/92) | Random/RR | 4.17 | 0.63, 27.71 | 80% | 0.14 | Not serious | Very serious ^c^ | Not serious | Serious ^d, e^ | NR | **⨁◯◯◯**  **Very low** |
| Yin(Yin et al., 2022) | Clinical improvement | UC | Curcumin/placebo | 5 | 296 (147/149) | Random/RR | 1.62 | 1.00, 2.61 | 64% | 0.05 | Not serious | Serious ^b^ | Not serious | Serious ^d, e^ | NR | **⨁◯◯◯**  **Very low** |
| Yin(Yin et al., 2022) | Endoscopic improvement | UC | Curcumin/placebo | 2 | 95(49/46) | Random/RR | 4.13 | 0.20, 87.07 | 79% | 0.36 | Not serious | Very serious ^c^ | Not serious | Serious ^d, e^ | NR | **⨁◯◯◯**  **Very low** |
| **Neuropsychiatric diseases** | | | | | | | | | | | | | | | | |
| Fathi(Fathi et al., 2024) | Anxiety symptoms | Anxiety | Curcumin/placebo | 8 | 567(NR) | Random/SMD | -1.56 | -2.48, -0.64 | 95.6% | < 0.001 | Serious ^a^ | Very serious ^c^ | Not serious | Not serious | NR | **⨁◯◯◯**  **Very low** |
| Wang(Wang et al., 2021b) | Depressive symptom*s* | Depression | Curcumin/placebo | 10 | 594(327/267) | Random/SMD | -0.32 | -0.50, -0.13 | 15% | 0.0007 | Serious ^a^ | Not serious | Not serious | Not serious | Not serious | **⨁⨁⨁◯**  **Moderate** |
| Wang(Wang et al., 2021b) | Response rates for Depressive symptoms | Depression | Curcumin/placebo | 3 | 271 (161/110) | Random/OR | 3.2 | 1.28, 7.99 | 35% | 0.01 | Serious ^a^ | Not serious | Not serious | Not serious | NR | **⨁⨁⨁◯**  **Moderate** |
| Sarraf(Sarraf et al., 2019) | BDNP level | Adults | Curcumin/placebo | 4 | 139 (69/70） | Random/WMD | 1789.38 | 722.04, 2856.71 | 83.5% | <0.01 | Serious ^a^ | Very serious ^c^ | Not serious | Serious ^e^ | Not serious | **⨁◯◯◯**  **Very low** |
| Zhu(Zhu et al., 2019) | Cognitive abilities | Old adults | Curcumin/placebo | 3 | 196 (90/106) | Random/SMD | 0.33 | 0.05,0.62 | 0% | 0.02 | Serious ^a^ | Not serious | Not serious | Serious ^e^ | NR | **⨁⨁◯◯**  **Low** |
| Zhu(Zhu et al., 2019) | Cognitive abilities | AD | Curcumin/placebo | 2 | 57 (38/19) | Random/SMD | -0.90 | -1.48, -0.32 | 0% | 0.002 | Serious ^a^ | Not serious | Not serious | Serious ^e^ | NR | **⨁⨁◯◯**  **Low** |
| Zhu(Zhu et al., 2019) | Depression | Old adults | Curcumin/placebo | 2 | 136 (60/76) | Random/SMD | −0.29 | -0.64,0.05 | 0% | 0.09 | Serious ^a^ | Not serious | Not serious | Serious ^e^ | NR | **⨁⨁◯◯**  **Low** |
| **Musculoskeletal Diseases** | | | | | | | | | | | | | | | | |
| Zeng(Zeng et al., 2021) | VAS score-pain | Osteoarthritis | Curcuma longa extract and curcumin/placebo | 6 | 381(216/165) | Random/WMD | -11.55, | -14.3, -9.06 | 69% | <0.000001 | Serious ^a^ | Serious ^b^ | Not serious | Not serious | Not serious | **⨁⨁◯◯**  **Low** |
| Zeng(Zeng et al., 2021) | VAS score-pain | Osteoarthritis | Curcuma longa extract and curcumin/ NSAIDs | 2 | 230(115/115) | Random/WMD | -0.34 | −1.25, 0.57 | 0% | 0.46 | Serious ^a^ | Not serious | Not serious | Very serious ^d, e^ | Not serious | **⨁◯◯◯**  **Very low** |
| Zeng(Zeng et al., 2021) | WOMAC score-pain | Osteoarthritis | Curcuma longa extract and curcumin/placebo | 4 | 315(157/158) | Random/WMD | -0.66 | -0.88, -0.43 | 34% | <0.00001 | Not serious | Not serious | Not serious | Not serious | Not serious | **⨁⨁⨁⨁**  **High** |
| Zeng(Zeng et al., 2021) | WOMAC score-function | Osteoarthritis | Curcuma longa extract and curcumin/placebo | 4 | 315(157/158) | Random/WMD | -0.79 | -1.27, -0.31 | 75% | 0.001 | Not serious | Very serious ^c^ | Not serious | Not serious | Not serious | **⨁⨁◯◯**  **Low** |
| Zeng(Zeng et al., 2021) | WOMAC score-stiffness | Osteoarthritiis | Curcuma longa extract and curcumin/placebo | 4 | 315(157/158) | Random/WMD | -0.35 | -0.57, -0.12 | 26% | 0.002 | Not serious | Not serious | Not serious | Not serious | Not serious | **⨁⨁⨁⨁**  **High** |
| Wang(Wang et al., 2021a) | Knee pain | Knee osteoarthritis | Turmeric Extracts /placebo | 12 | 1071(577/494) | Random/SMD | -0.82 | - 1.17, - 0.47 | 86.23% | 0.00 | Serious ^a^ | Very serious ^c^ | Not serious | Not serious | Serious ^f^ | **⨁◯◯◯**  **Very low** |
| Wang(Wang et al., 2021a) | Knee pain | Knee osteoarthritis | Turmeric Extracts /NSAIDs | 5 | 648(342/306) | Random/SMD | -0.09 | -0.30, 0.12 | 34.97% | 0.2 | Serious ^a^ | Not serious | Not serious | Serious ^d,^ | Serious ^f^ | **⨁◯◯◯**  **Very low** |
| Wang(Wang et al., 2021a) | Physical function | Knee osteoarthritis | Turmeric Extracts /placebo | 10 | 973(508/465) | Random/SMD | -0.75 | -1.18, -0.33 | 90.05% | 0.00 | Serious ^a^ | Very serious ^c^ | Not serious | Not serious | Serious ^f^ | **⨁◯◯◯**  **Very low** |
| Wang(Wang et al., 2021a) | Physical function | Knee osteoarthritis | Turmeric Extracts / NSAIDs | 3 | 477(258/219) | Random/SMD | -0.14 | -0.36, 0.09 | 20.02% | 0.35 | Serious ^a^ | Not serious | Not serious | Serious ^d^ | Serious ^f^ | **⨁◯◯◯**  **Very low** |
| Beba(Beba et al., 2022) | CK level | Adults | Curcumin/placebo | 9 | NR | Random/WMD | -65.98 IU/ | -99.53, -32.44 | 86.8% | 0.000 | Serious ^a^ | Very serious ^c^ | Not serious | Serious ^e^ | Not serious | **⨁◯◯◯**  **Very low** |
| Beba(Beba et al., 2022) | Muscle soreness | Adults | Curcumin/placebo | 10 | NR | Random/WMD | -0.56 | -0.84, -0.27 | 61.2% | 0.000 | Serious ^a^ | Serious ^b^ | Not serious | Not serious | Not serious | **⨁⨁◯◯**  **Low** |
| Beba(Beba et al., 2022) | Muscle strength | Adults | Curcumin/placebo | 3 | NR | Random/WMD | 3.10nm | 1.45, 4.75 | 0% |  | Serious ^a^ | Not serious | Not serious | Serious ^e^ | Not serious | **⨁⨁◯◯**  **Low** |
| Beba(Beba et al., 2022) | Joint flexibility | Adults | Curcumin/placebo | 3 | NR | Random/WMD | 6.49° | 3.91, 9.07 | 71.7% |  | Serious ^a^ | Serious ^b^ | Not serious | Serious ^e^ | Not serious | **⨁◯◯◯**  **Very low** |
| **Liver and kidney diseases** | | | | | | | | | | | | | | | | |
| Malekmakan(Malekmakan et al., 2022) | Proteinuria level | CKD | Curcumin/placebo | 4 | 265(138/127) | Fixed/SMD | -0.72 | -1.10，-0.35 | 46.2% | 0.003 | Serious ^a^ | Not serious | Not serious | Not serious | Not serious | **⨁⨁⨁◯**  **Moderate** |
| Dehzad(Dehzad et al., 2023b) | ALT level | Adults | Curcumin/placebo | 31 | NR | Random/WMD | -4.09 U/L | -6.49, -1.70 | 95.8% | 0.000 | Serious ^a^ | Very serious ^c^ | Not serious | Not serious | Not serious | **⨁◯◯◯**  **Very low** |
| Dehzad(Dehzad et al., 2023b) | AST level | Adults | Curcumin/placebo | 29 | NR | Random/WMD | -3.81U/L | -5.71, -1.91 | 96.3% | 0.000 | Serious ^a^ | Very serious ^c^ | Not serious | Not serious | Not serious | **⨁◯◯◯**  **Very low** |
| Dehzad(Dehzad et al., 2023b) | GGT level | Adults | Curcumin/placebo | 5 | NR | Random/WMD | -12.78U/L | -28.20,2.64 | 98.0% | 0.000 | Serious ^a^ | Very serious ^c^ | Not serious | Not serious | Not serious | **⨁◯◯◯**  **Very low** |
| **Gynaecological diseases** | | | | | | | | | | | | | | | | |
| Sharifipour(Sharifipour et al., 2024) | Dysmenorrhea severity | Reproductive-aged women | Curcumin/placebo | 3 | 253(124/129) | Fixed/MD | -1.25 | -1.52, -0.98 | 31% | <0.00001 | Not serious | Not serious | Not serious | Serious ^e^ | Not serious | **⨁⨁⨁◯**  **Moderate** |
| Sharifipour(Sharifipour et al., 2024) | PMS severity | Reproductive-aged women | Curcumin/placebo | 3 | 244(121/123) | Fixed/SMD | -0.73 | -1.00, -0.47 | 91% | <0.00001 | Not serious | Very serious ^c^ | Not serious | Serious ^e^ | Not serious | **⨁⨁◯◯**  **Low** |
| Sharifipour(Sharifipour et al., 2024) | Behavioral symptoms of PMS | Reproductive-aged women | Curcumin/placebo | 2 | 126(64/62) | Fixed/MD | -12.90 | -17.82, -7.99 | 0% | <0.00001 | Not serious | Not serious | Not serious | Serious ^e^ | Not serious | **⨁⨁⨁◯**  **Moderate** |
| Sharifipour(Sharifipour et al., 2024) | Mood Symptoms of PMS | Reproductive-aged women | Curcumin/placebo | 2 | 126(64/62) | Fixed/MD | -17.61 | -22.75, -12.46 | 0% | <0.00001 | Not serious | Not serious | Not serious | Serious ^e^ | Not serious | **⨁⨁⨁◯**  **Moderate** |
| Sharifipour(Sharifipour et al., 2024) | Physical symptoms of PMS | Reproductive-aged women | Curcumin/placebo | 2 | 126(64/62) | Fixed/MD | -19.65 | -25.50, -13.80 | 0% | <0.00001 | Not serious | Not serious | Not serious | Serious ^e^ | Not serious | **⨁⨁⨁◯**  **Moderate** |
| Shen(Shen et al., 2022) | Testosterone | PCOS | Curcumin/placebo, medication | 2 | NR | Random/WMD | -0.128 | -0.383, 0.127 | 98.6% | 0.326 | Serious ^a^ | Very serious ^c^ | Not serious | Very serious ^d,^ e | NR | **⨁◯◯◯**  **Very low** |
| Shen(Shen et al., 2022) | DHEA | PCOS | Curcumin/placebo, medication | 3 | NR | Random/WMD | -8.239 | -30.260, 13.781 | 62.3% | 0.463 | Serious ^a^ | Serious ^b^ | Not serious | Very serious ^d,^ e | NR | **⨁◯◯◯**  **Very low** |
| Shen(Shen et al., 2022) | LH | PCOS | Curcumin/placebo, medication | 3 | NR | Random/WMD | -0.003 | -0.007, 0.000 | 0.0% | 0.087 | Serious ^a^ | Not serious | Not serious | Very serious ^d,^ e | NR | **⨁◯◯◯**  **Very low** |
| Shen(Shen et al., 2022) | FSH | PCOS | Curcumin/placebo, medication | 3 | NR | Random/WMD | 0.002 | -0.024, 0.029 | 0.0% | 0.854 | Serious ^a^ | Not serious | Not serious | Very serious ^d,^ e | NR | **⨁◯◯◯**  **Very low** |
| Shen(Shen et al., 2022) | LH/FSH | PCOS | Curcumin/placebo, medication | 3 | NR | Random/WMD | -0.114 | -0.311, 0.084 | 0.0% | 0.259 | Serious ^a^ | Not serious | Not serious | Very serious ^d,^ e | NR | **⨁◯◯◯**  **Very low** |
| Shen(Shen et al., 2022) | FAI | PCOS | Curcumin/placebo, medication | 2 | NR | Random/WMD | -0.245 | -1.138 to 0.647 | 30.0% | 0.590 | Serious ^a^ | Not serious | Not serious | Very serious ^d,^ e | NR | **⨁◯◯◯**  **Very low** |
| **Radiation Dermatitis** | | | | | | | | | | | | | | | | |
| Dahka(Mirzaei Dahka et al., 2023) | RDS | Breast cancer | Curcumin/placebo | 4 | 882(NR) | Random/WMD | -0.50 | -0.72, -0.27 | 95.7% | <0.001 | Serious ^a^ | Very serious ^c^ | Not serious | Not serious | Not serious | **⨁◯◯◯**  **Very low** |
| **Pain** | | | | | | | | | | | | | | | | |
| Sahebkar(Sahebkar and Henrotin, 2016) | Pain severity | NA | Curcuminoids/control | 8 | 606(306/300) | Random/SMD | -0.57 | -1.1，-0.03 | 86% | 0.04 | Serious ^a^ | Very serious ^c^ | Not serious | Not serious | Serious | **⨁◯◯◯**  **Very low** |
| **HRQOL** | | | | | | | | | | | | | | | | |
| Sadeghian(Sadeghian et al., 2021) | HRQOL | NA | Curcumin/placebo | 10 | 730(355/375) | Random/SMD | 2.46 | 1.30,3.63 | 97.4 | <0.01 | Serious ^a^ | Very serious ^c^ | Not serious | Not serious | Serious | **⨁◯◯◯**  **Very low** |
| **COVID-19** | | | | | | | | | | | | | | | | |
| Shafiee(Shafiee et al., 2023) | All-cause mortality | COVID-19 | Curcumin/placebo or standard of care | 7 | 665(334/331) | Random/RR | 0.37 | 0.21,0.65 | 0% | 0.0005 | Serious ^a^ | Not serious | Not serious | Not serious | NR | **⨁⨁⨁◯**  **Moderate** |
| Shafiee(Shafiee et al., 2023) | Incidence of mechanical ventilation | COVID-19 | Curcumin/placebo or standard of care | 3 | 386(192/194) | Random/RR | 0.23 | 0.05,1.07 | 0% | 0.06 | Not serious | Not serious | Not serious | Serious ^d^ | NR | **⨁⨁⨁◯**  **Moderate** |
| Shafiee(Shafiee et al., 2023) | Incidence of hospitalization | COVID-19 | Curcumin/placebo or standard of care | 2 | 152(75/77) | Random/RR | 0.17 | 0.02,1.4 | 0% | 0.1 | Not serious | Not serious | Not serious | Very serious ^d, e^ | NR | **⨁⨁◯◯**  **Low** |
| Shafiee(Shafiee et al., 2023) | Positive RT-PCR rate | COVID-19 | Curcumin/placebo or standard of care | 3 | 160(88/72) | Random/RR | 0.55 | 0.4,0.77 | 32% | 0.0004 | Serious ^a^ | Not serious | Not serious | Serious ^d^ | NR | **⨁⨁◯◯**  **Low** |
| Shafiee(Shafiee et al., 2023) | Rate of no recovery | COVID-19 | Curcumin/placebo or standard of care | 7 | 509(257/252) | Random/RR | 0.55 | 0.43.0.69 | 0% | <0.0001 | Serious ^a^ | Not serious | Not serious | Not serious | NR | **⨁⨁⨁◯**  **Moderate** |
| **Rheumatoid arthritis** | | | | | | | | | | | | | | | | |
| Kou(Kou et al., 2023) | VAS pain | RA | Curcumin/placebo or standard of care | 3 | 66 (34/32) | Random/MD | -5.32 | -9.42, -1.22 | 19% | 0.01 | Serious ^a^ | Not serious | Not serious | Serious ^e^ | Not serious | **⨁⨁◯◯**  **Low** |
| Kou(Kou et al., 2023) | DAS28 | RA | Curcumin/placebo or standard of care | 6 | 273(137/136) | Random/MD | -1.20 | -1.85, -0.55 | 92% | 0.0003 | Serious ^a^ | Very serious ^c^ | Not serious | Serious ^e^ | Not serious | **⨁◯◯◯**  **Very low** |
| Kou(Kou et al., 2023) | Tender joint count | RA | Curcumin/placebo or standard of care | 4 | 119(59/60) | Random/MD | -6.33 | -10.86, -1.81 | 98% | 0.0006 | Serious ^a^ | Very serious ^c^ | Not serious | Serious ^e^ | Not serious | **⨁◯◯◯**  **Very low** |
| Kou(Kou et al., 2023) | Swollen joint count | RA | Curcumin/placebo or standard of care | 4 | 119(59/60) | Random/MD | -5.33 | -9.90, -0.76 | 98% | 0.02 | Serious ^a^ | Very serious ^c^ | Not serious | Serious ^e^ | Not serious | **⨁◯◯◯**  **Very low** |
| Kou(Kou et al., 2023) | RF | RA | Curcumin/placebo or standard of care | 4 | 153(77/76) | Random/MD | -24.15 | -36.47, -11.83 | 97% | 0.0001 | Serious ^a^ | Very serious ^c^ | Not serious | Serious ^e^ | Not serious | **⨁◯◯◯**  **Very low** |
| Kou(Kou et al., 2023) | ACR20 | RA | Curcumin/placebo or standard of care | 2 | 68(34/34) | Random/MD | 0.96 | 0.39,1.52 | 0% | 0.00009 | Serious ^a^ | Not serious | Not serious | Serious ^e^ | Not serious | **⨁⨁◯◯**  **Low** |

Abbreviations: MD: mean difference; SMD standard mean difference; RR: relative risk; OR: odd risk; NR: not reported; I: intervention; C: comparator; T2DM, Type 2 diabetes mellitus; HbA1c, hemoglobin A1c; NAFLD, nonalcoholic fatty liver disease; FBG: fasting blood glucose; HOMA-IR: homeostatic model assessment for insulin resistance; QUICKI: quantitative insulin-sensitivity check index; PCOS: polycystic ovarian syndrome; INS: insulin; OGTT: oral glucose tolerance test; SBP: systolic blood pressure; DBP: diastolic blood pressure; VCAM-1: vascular cell adhesion molecule-1; FMD: flow-mediated dilation; PWV: pulse wave velocity ; TC: total cholesterol; TG:  triglyceride; LDL-C: low-density-lipoprotein cholesterol; HDL-C: high-density lipoprotein cholesterol; Apo-A: apolipoproteins A; Apo-B: apolipoproteins B; BMI: body mass index ; CRP: C-reactive protein; TNF-α: tumor necrosis factor-alpha; IL-6: interleukin-6; IL-1β: interleukin 1beta; IL-8: interleukin-8; RA : rheumatoid arthritis; TAC:  total antioxidant capacity; MDA: malondialdehyde; SOD: superoxide dismutase; IBS: irritable bowel syndrome; BDNF: brain-derived neurotrophic factor; WOMAC: Western Ontario and McMaster Universities Osteoarthritis Index; VAS: visual analogue scale; ALT: alanine aminotransferase; AST: aspartate aminotransferase; GGT: gamma-glutamyltransferase; DHEA: dehydroepiandrosterone-sulfate; LH: luteinizing hormone; FSH: follicle-stimulating hormone; FAI: free androgen index; PMS: premenstrual syndrome

◯ represented “low”; ⨁ represented “high”.

The GRADE approach provides four levels of certainty for the estimated effect: High: The quality of evidence is high. Further research is very unlikely to change the confidence level.; Moderate: The quality of evidence is moderate. Further research may change the confidence level; Low: The quality of evidence is low. Further research is likely to change the confidence level; Very low: The quality of evidence is very low.

**References:**

Beba, M., Mohammadi, H., Clark, C.C.T., and Djafarian, K. (2022). The effect of curcumin supplementation on delayed-onset muscle soreness, inflammation, muscle strength, and joint flexibility: A systematic review and dose-response meta-analysis of randomized controlled trials. *Phytotherapy Research : PTR* 36(7)**,** 2767-2778. doi: 10.1002/ptr.7477.

Dehzad, M.J., Ghalandari, H., Amini, M.R., and Askarpour, M. (2023a). Effects of curcumin/turmeric supplementation on lipid profile: A GRADE-assessed systematic review and dose-response meta-analysis of randomized controlled trials. *Complementary Therapies In Medicine* 75**,** 102955. doi: 10.1016/j.ctim.2023.102955.

Dehzad, M.J., Ghalandari, H., Amini, M.R., and Askarpour, M. (2023b). Effects of curcumin/turmeric supplementation on liver function in adults: A GRADE-assessed systematic review and dose-response meta-analysis of randomized controlled trials. *Complementary Therapies In Medicine* 74**,** 102952. doi: 10.1016/j.ctim.2023.102952.

Dehzad, M.J., Ghalandari, H., and Askarpour, M. (2024). Curcumin/turmeric supplementation could improve blood pressure and endothelial function: A grade-assessed systematic review and dose-response meta-analysis of randomized controlled trials. *Clinical Nutrition ESPEN* 59**,** 194-207. doi: 10.1016/j.clnesp.2023.12.009.

Dehzad, M.J., Ghalandari, H., Nouri, M., and Askarpour, M. (2023c). Antioxidant and anti-inflammatory effects of curcumin/turmeric supplementation in adults: A GRADE-assessed systematic review and dose-response meta-analysis of randomized controlled trials. *Cytokine* 164**,** 156144. doi: 10.1016/j.cyto.2023.156144.

Dehzad, M.J., Ghalandari, H., Nouri, M., and Askarpour, M. (2023d). Effects of curcumin/turmeric supplementation on obesity indices and adipokines in adults: A grade-assessed systematic review and dose-response meta-analysis of randomized controlled trials. *Phytotherapy Research : PTR* 37(4)**,** 1703-1728. doi: 10.1002/ptr.7800.

Ebrahimzadeh, A., Mohseni, S., Safargar, M., Mohtashamian, A., Niknam, S., Bakhoda, M., et al. (2024). Curcumin effects on glycaemic indices, lipid profile, blood pressure, inflammatory markers and anthropometric measurements of non-alcoholic fatty liver disease patients: A systematic review and meta-analysis of randomized clinical trials. *Complementary Therapies In Medicine* 80**,** 103025. doi: 10.1016/j.ctim.2024.103025.

Emami, E., Heidari-Soureshjani, S., and Sherwin, C.M. (2022). Anti-inflammatory response to curcumin supplementation in chronic kidney disease and hemodialysis patients: A systematic review and meta-analysis. *Avicenna Journal of Phytomedicine* 12(6)**,** 576-588. doi: 10.22038/AJP.2022.20049.

Fathi, S., Agharloo, S., Falahatzadeh, M., Bahraminavid, S., Homayooni, A., Faghfouri, A.H., et al. (2024). Effect of curcumin supplementation on symptoms of anxiety: A systematic review and meta-analysis of randomized controlled trials. *Clinical Nutrition ESPEN* 62**,** 253-259. doi: 10.1016/j.clnesp.2024.05.017.

Kou, H., Huang, L., Jin, M., He, Q., Zhang, R., and Ma, J. (2023). Effect of curcumin on rheumatoid arthritis: a systematic review and meta-analysis. *Frontiers In Immunology* 14**,** 1121655. doi: 10.3389/fimmu.2023.1121655.

Malekmakan, L., Hamidianjahromi, A., Sayadi, M., and Rezazadeh, M.H. (2022). Efficacy and Safety of Turmeric Dietary Supplementation on Proteinuria in CKD: A Systematic Review and Meta-analysis of RCT. *Iranian Journal of Kidney Diseases* 16(3)**,** 153-161.

Mirzaei Dahka, S., Afsharfar, M., Tajaddod, S., Sohouli, M.H., Shekari, S., Bakhshi Nafouti, F., et al. (2023). Impact of Curcumin Supplementation on Radiation Dermatitis Severity: A Systematic Review and Meta-Analysis of Randomized Controlled Trials. *Asian Pacific Journal of Cancer Prevention : APJCP* 24(3)**,** 783-789. doi: 10.31557/APJCP.2023.24.3.783.

Ng, Q.X., Soh, A.Y.S., Loke, W., Venkatanarayanan, N., Lim, D.Y., and Yeo, W.-S. (2018). A Meta-Analysis of the Clinical Use of Curcumin for Irritable Bowel Syndrome (IBS). *Journal of Clinical Medicine* 7(10). doi: 10.3390/jcm7100298.

Sadeghian, M., Rahmani, S., Jamialahmadi, T., Johnston, T.P., and Sahebkar, A. (2021). The effect of oral curcumin supplementation on health-related quality of life: A systematic review and meta-analysis of randomized controlled trials. *Journal of Affective Disorders* 278**,** 627-636. doi: 10.1016/j.jad.2020.09.091.

Sahebkar, A., and Henrotin, Y. (2016). Analgesic Efficacy and Safety of Curcuminoids in Clinical Practice: A Systematic Review and Meta-Analysis of Randomized Controlled Trials. *Pain Medicine (Malden, Mass.)* 17(6)**,** 1192-1202. doi: 10.1093/pm/pnv024.

Sarraf, P., Parohan, M., Javanbakht, M.H., Ranji-Burachaloo, S., and Djalali, M. (2019). Short-term curcumin supplementation enhances serum brain-derived neurotrophic factor in adult men and women: a systematic review and dose-response meta-analysis of randomized controlled trials. *Nutrition Research (New York, N.Y.)* 69**,** 1-8. doi: 10.1016/j.nutres.2019.05.001.

Shafiee, A., Athar, M.M.T., Shahid, A., Ghafoor, M.S., Ayyan, M., Zahid, A., et al. (2023). Curcumin for the treatment of COVID-19 patients: A meta-analysis of randomized controlled trials. *Phytotherapy Research : PTR* 37(3)**,** 1167-1175. doi: 10.1002/ptr.7724.

Sharifipour, F., Siahkal, S.F., Qaderi, K., Mohaghegh, Z., Zahedian, M., and Azizi, F. (2024). Effect of Curcumin on Dysmenorrhea and Symptoms of Premenstrual Syndrome: A Systematic Review and Meta-Analysis. *Korean Journal of Family Medicine* 45(2). doi: 10.4082/kjfm.23.0184.

Shen, W., Qu, Y., Jiang, H., Wang, H., Pan, Y., Zhang, Y., et al. (2022). Therapeutic effect and safety of curcumin in women with PCOS: A systematic review and meta-analysis. *Frontiers In Endocrinology* 13**,** 1051111. doi: 10.3389/fendo.2022.1051111.

Tian, J., Feng, B., and Tian, Z. (2022). The Effect of Curcumin on Lipid Profile and Glycemic Status of Patients with Type 2 Diabetes Mellitus: A Systematic Review and Meta-Analysis. *Evidence-based Complementary and Alternative Medicine : ECAM* 2022**,** 8278744. doi: 10.1155/2022/8278744.

Wang, Z., Singh, A., Jones, G., Winzenberg, T., Ding, C., Chopra, A., et al. (2021a). Efficacy and Safety of Turmeric Extracts for the Treatment of Knee Osteoarthritis: a Systematic Review and Meta-analysis of Randomised Controlled Trials. *Current Rheumatology Reports* 23(2)**,** 11. doi: 10.1007/s11926-020-00975-8.

Wang, Z., Zhang, Q., Huang, H., and Liu, Z. (2021b). The efficacy and acceptability of curcumin for the treatment of depression or depressive symptoms: A systematic review and meta-analysis. *Journal of Affective Disorders* 282**,** 242-251. doi: 10.1016/j.jad.2020.12.158.

Yin, J., Wei, L., Wang, N., Li, X., and Miao, M. (2022). Efficacy and safety of adjuvant curcumin therapy in ulcerative colitis: A systematic review and meta-analysis. *Journal of Ethnopharmacology* 289**,** 115041. doi: 10.1016/j.jep.2022.115041.

Zeng, L., Yu, G., Hao, W., Yang, K., and Chen, H. (2021). The efficacy and safety of Curcuma longa extract and curcumin supplements on osteoarthritis: a systematic review and meta-analysis. *Bioscience Reports* 41(6). doi: 10.1042/BSR20210817.

Zhu, L.-N., Mei, X., Zhang, Z.-G., Xie, Y.-P., and Lang, F. (2019). Curcumin intervention for cognitive function in different types of people: A systematic review and meta-analysis. *Phytotherapy Research : PTR* 33(3)**,** 524-533. doi: 10.1002/ptr.6257.
